# Degradation of sexual reproduction in *Veronica filiformis* after introduction to Europe

**DOI:** 10.1186/1471-2148-12-233

**Published:** 2012-12-03

**Authors:** Romain Scalone, Dirk C Albach

**Affiliations:** 1 Institut für Spezielle Botanik und Botanischer Garten, Johannes Gutenberg-Universität Mainz, Bentzelweg 9, Mainz, 55099, Germany; 2 Department of Crop Production Ecology, Swedish University of Agricultural Science, Box 7043, Ulls väg 16, Uppsala, 75007, Sweden; 3 Institut für Biologie und Umweltwissenschaften (IBU), Carl von Ossietzky-Universität Oldenburg, Carl von Ossietzky-Str. 9-11, Oldenburg, 26111, Germany

## Abstract

**Background:**

Baker’s law predicts that self-incompatible plant species are generally poor colonizers because their mating system requires a high diversity of genetically differentiated individuals and thus self-compatibility should develop after long-distance dispersal. However, cases like the introduction of the self-incompatible *Veronica filiformis* (Plantaginaceae) to Europe constitute an often overlooked alternative to this rule. This species was introduced from subalpine areas of the Pontic-Caucasian Mountains and colonized many parts of Central and Western Europe in the last century, apparently without producing seeds. To investigate the consequences of the absence of sexual reproduction in this obligate outcrosser since its introduction, AFLP fingerprints, flower morphology, pollen and ovule production and seed vitality were studied in introduced and native populations.

**Results:**

Interpopulation crossings of 19 introduced German populations performed in the greenhouse demonstrated that introduced populations are often unable to reproduce sexually. These results were similar to intrapopulation crossings, but this depended on the populations used for crossings. Results from AFLP fingerprinting confirmed a lack of genetic diversity in the area of introduction, which is best explained by the dispersal of clones. Flower morphology revealed the frequent presence of mutations affecting the androecium of the flower and decreasing pollen production in introduced populations. The seeds produced in our experiments were smaller, had a lower germination rate and had lower viability than seeds from the native area.

**Conclusions:**

Taken together, our results demonstrate that *V. filiformis* was able to spread by vegetative means in the absence of sexual reproduction. This came at the cost of an accumulation of phenotypically observable mutations in reproductive characters, i.e. Muller’s ratchet.

## Background

Humans are changing their environment in hitherto unprecedented ways. One aspect of change is the introduction of organisms to new environments, thus creating many evolutionary “experiments”. While most of these introductions fail [1], those that are successful are often the source of economic and ecological problems [2]. For evolutionary biologists, introductions can also be important to understand processes of evolution. In particular, mating system evolution of newly founded populations is a highly relevant aspect of introductions [3,4]. Based on observations of plant species dispersed to islands, Baker [5] concluded that successful colonizers have a higher probability of being self-compatible (SC) and capable of autonomous self-pollination. This capacity is an advantage during the establishment of populations from a single or very few individuals in a new environment where the chance of encountering a mating partner or an efficient pollinator is reduced. This observation has subsequently been termed “Baker’s rule” [6,7]. Although Baker neglected other important factors favoring or limiting the evolution of selfing [8], his idea has been a useful initial hypothesis for many studies on colonizing species. Recent theoretical studies have demonstrated that self-incompatible (SI) species can become successful colonisers via evolution of the mating system and/or dispersal rate [9]. If dispersal to compatible mating partners is not possible, SI species can establish themselves either via selection for selfing and breakdown of their SI system, or by relying on vegetative reproduction and longevity [10-13]. Both strategies have essentially the same consequence, i.e. a decrease in genetic diversity. Further reduction of genetic diversity occurs in bottlenecks caused by small population size of the initial colonizing population (“founder effect”). Whether high genetic diversity in introduced plant populations is required for successful invasion has been a matter of debate for some time [14-16] and is apparently not necessary in all cases [17,18]. In the extreme case, single clones have been shown to invade all of Europe [14,19,20], although more and more studies have detected high genetic variation even in clonal invasive plants [21,22]. Nevertheless, the origin of this diversity is mostly unknown. Thus, successful clonal invaders are either optimally pre-adapted to the environment encountered, constitute a “general-purpose genotype” sensu Baker [5] or they are able to adapt to local conditions without large amounts of genetic diversity.

The neglible influence of low genetic diversity on the ability to adapt to local conditions has been demonstrated in meta-analyses, which also indicated that small population size has an impact [23,24]. SI species have the further disadvantage in colonizing situations in that the small initial, introduced population may be subject to the Allee effect, i.e. the low success of outcrossing at low density of compatible individuals, which possibly drives the population to extinction [25,26].

Whereas overall genetic diversity seems to be inconsequential for SI colonizing species able to reproduce vegetatively, genetic diversity at the S-locus responsible for the SI reaction is necessary for sexual reproduction in SI species. For such a species a large number of S-alleles in each population (~30-40; [27]) is required for the maintenance of an SI system in a natural population. Although exceptions exist, an introduced population is often founded by few individuals that are genetically closely related and therefore share a low and limited number of S-alleles [28]. In that case, after the first few generations, all individuals belonging to the new population likely share the same S-alleles due to inbreeding, and sexual reproduction becomes impossible [29]. To restore sexual reproduction, an increase in genetic diversity is necessary and can be achieved in several ways, i.e. high dispersal rate and merging of previously separated populations, multiple introductions of the invasive species [30], somatic mutations [31] or hybridization with local relatives [32,33]. Whereas all mechanisms have been demonstrated to increase overall genetic diversity, none has yet been shown to increase S-allele diversity. If overall genetic diversity is increased by mutations, these are considered to be predominantly deleterious [34,35] and the lack of sexual reproduction leads to their accumulation with subsequent detrimental effect (“Muller’s ratchet”; [36]). Mildly deleterious mutations are certain to accumulate more rapidly than strongly deleterious mutations and thus mutations should accumulate predominantly in genes underlying traits which no longer enhance fitness rather than in those which are important for fitness [12]. In clonal plants, loss of sexual reproduction reduces fitness in the long term, because it prevents purging of mutation load following recombination. However, in the short term, fitness can be increased if resources are diverted from flowers to vegetative growth [37]. The effect of mutations on the S-locus is unknown. The most likely outcome is the disruption of the SI system, which may nevertheless be just an SC intermediate in the path to a new S-allele [38]. Understanding trade-offs between clonal and sexual reproduction, and how they are influenced by dispersal, is important for evaluating the survival probability of clonally reproducing SI species and their future invasion potential [13,17].

**Figure 1:**
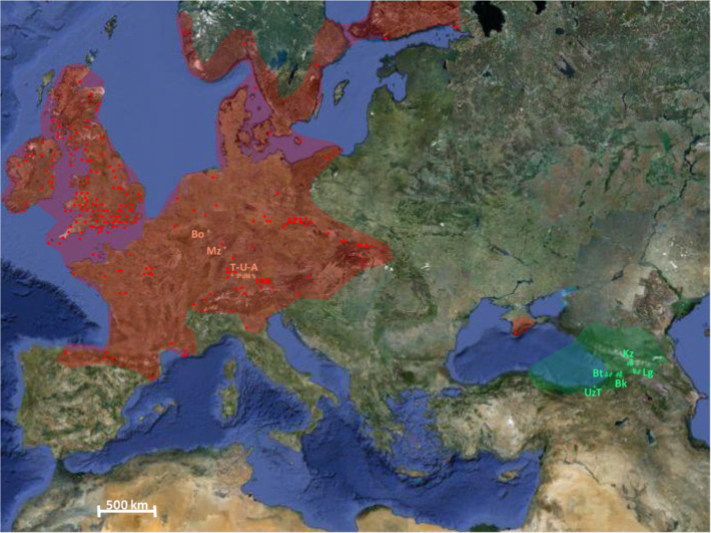
**Distribution of *V****. ****filiformis *in the native** (**green zone**) **and introduced** (**red zone**) **areas.** The red points correspond to the introduced populations of *V. filiformis* mentioned in the references given in the introduction. The orange and green crosses correspond to the populations used during this study from the introduced and the native areas, respectively. Bk = Bakuriani (Samtskhe-Javakhéti region), Bo = Bonn (North Rhine-Westphalia), Bt = Batumi (Adjara region), Kz = Kazbegi (Kazbegi region), Lg = Lagodekhi (Kakheti region), Mz = Mainz (Rhineland-Palatinate), T-U-A = transect Tübingen-Ulm-Augsburg, (Baden-Württemberg and Bavaria), UzT = Uzungöl-Trabzon (Trabzon province).

One model to address these questions is the Pontic-Anatolian-Caucasian *Veronica filiformis* (Figure 1), an obligate self-incompatible (SI) and perennial species [39-41]. The first European records of *V. filiformis* came from Great Britain (1780, 1838) but it was not recorded again there until 1927 [42,43]. In mainland Europe, the first record (1893, Marseille, southern France) is associated with plants being packed around the roots of vine shoots imported from Georgia [44]. From that time, the history of introduction in the rest of Europe through horticultural trade is fairly well-known starting in Switzerland (1903), other parts of France (1904), Germany (Tübingen 1909; München 1923; Ulm 1936, Augsburg 1939), Great Britain, Austria, the Netherlands and then other regions of Europe [40,43-45] Figure 1. Most European populations of this species are described as sterile since no seed production was observed in the introduced area [39,42].

In the absence of sexual reproduction, *V. filiformis* seems to have colonized Europe by vegetative propagation favoured by several traits of the species. First, the species easily produces abundant adventitious roots on each node of the stem, which facilitates vegetative reproduction by fragmentation via cutting, mowing, raking or grazing [46,47]. Indeed, only three days after cutting, almost all nodes of each new fragment of this creeping species produce adventitious roots [47,48] and pers. obs. and facilitate the survival of the new “individuals”. Second, a single individual of *V. filiformis* can spread over more than 40 cm^2^ per year and produce dozens of new fragments with almost no mortality under standard conditions in the introduced area [49] and pers. obs. Optimal growth is achieved under low summer temperatures with high rainfall [49] in ecosystems such as pasture lands, lawns and turfs [50,51]. In these human-made habitats, *V. filiformis* can easily be dispersed by humans and their machines, animals or water depending on the environment [49], spread over several kilometres a year [52-54], and infest these habitats by covering up to 80-90% of the grassed area [55].

The species may therefore offer a suitable subject for both the mid- to long-term consequences of strict vegetative reproduction, as well as a model for the colonizing abilities of clonally reproducing species. At the moment, however, little is known about the genetic diversity, the distribution of clones, the mating system and dispersal in introduced populations of *V. filiformis*. For these reasons, the objectives of this study were to (1) determine the genetic diversity within and among populations in the area of introduction, (2) test the potential for sexual reproduction in Europe, (3) infer the importance of clonal dispersal and finally, (4) evaluate the impact of colonization on the reproductive potential of *V. filiformis*. For this purpose, intra- and inter-population crossing experiments were conducted along a regional transect of twenty German populations (~150 km) in the area of first introductions in southern Germany (between Tübingen, Baden-Württemberg “BW”, and Augsburg, Bavaria “BV”; T-U-A in Figure 1), followed by AFLP-fingerprinting of these German populations and native populations from Georgia and Turkey. These experiments permitted the definition of crossing groups (i.e., one crossing group is composed of individuals that never produce any seeds when crossed with each other, and which are, therefore, inferred to have the same SI alleles) and clones (i. e., two specimens are considered as clones when their rate of pairwise individual comparisons is below the error rate of the AFLP analysis) as well as an estimation of mate availability within this region of southern Germany. Flower morphology, number of pollen and ovules, seed number and size, germination rate and seed viability were measured on several populations from the introduced and native areas to determine the impact of lacking sexual reproduction on the reproductive potential of *V. filiformis*.

## Results

### Crossing experiments

A total of 436 flowers from 145 specimens were crossed in our intra- and interpopulation crossing experiments with a median number of five flowers per specimen (minimum 2, maximum 18). Forty-one flowers from eight specimens of the Pliezhausen (Pl) population did not produce any capsules after intra-population crossings.

**Figure 2:**
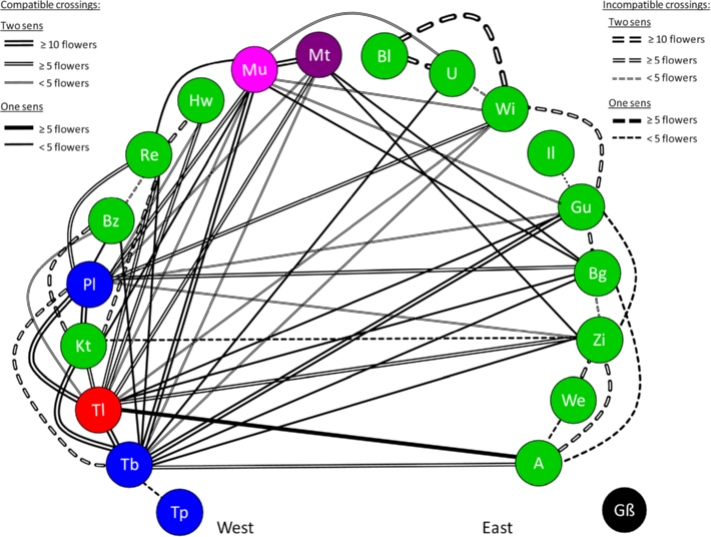
**Crossing polygon indicating results from interpopulation crossings.** The color of the circles corresponds to the crossing groups inferred by the crossing experiment. Names of the populations correspond to the codes in Table 1. A double line indicates that the crossings were done in both directions (♀ pop. X with ♂ pop. Y as well as ♂ pop X with ♀pop. Y), whereas unique single line means that only one direction of the cross was conducted. The width of the lines is proportional to the number of flowers tested for this crossing. The dashed lines refer to incompatible crossings, whereas solid lines indicate compatible crossings leading to the formation of seeds.

Populations were assigned to crossing groups when crossings between populations did not produce seeds (dashed lines; Figure 2) but did so when crossed with populations from other crossing groups (solid line; Figure 2). By this method, 19 populations were assigned to five different crossing groups represented by different colors (with non-capitalized color names) in Figure 2.

The largest crossing group consisted of thirteen populations from Baden-Württemberg to Bavaria (green colour, Figure 2). Three populations from the most western part of our transect (Tübingen private garden (Tp), Tübingen old botanical garden (Tb), and Pl) formed a second crossing group (blue color, Figure 2). The other three populations formed crossing groups of their own: Tübingen Lustnau (Tl, red, Figure 2), Münsingen (Mü, pink, Figure 2) and Mehrstetten (Mt, violet, Figure 2). The Großaitingen (Gß) population could not be grouped since individuals never produced flowers in the greenhouse and no flower was observed in the field during the two collecting trips.

### AFLP results

#### Population structure

**Figure 3:**
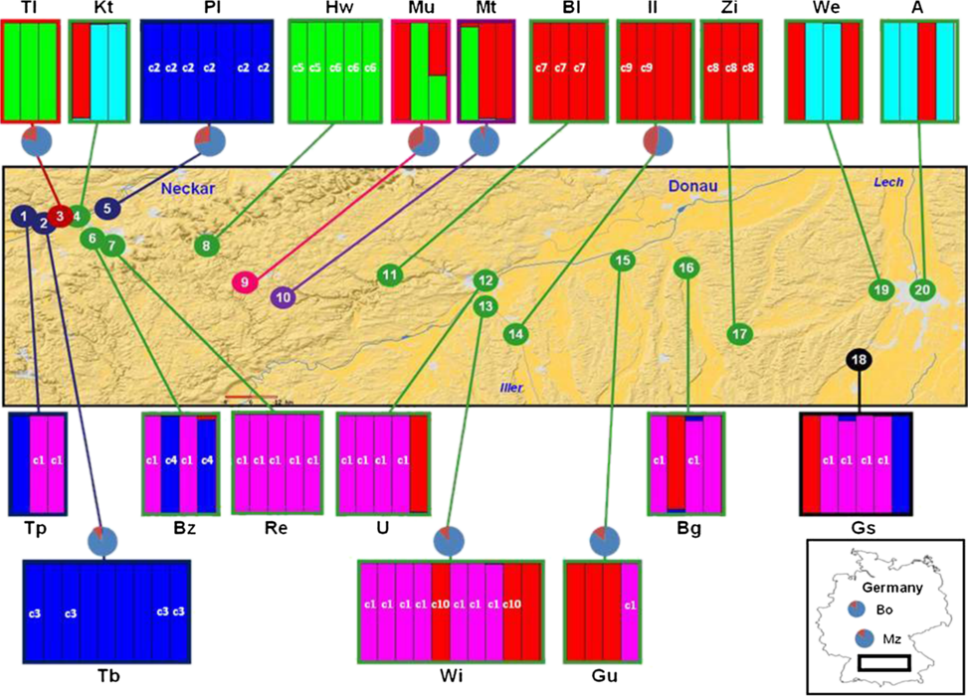
**Map of the transect.** The color of the localities within the map corresponds to the crossing groups of populations determined by the crossing experiment (Figure 2). The colored bars outside the map refer to the genetic clusters inferred by STRUCTURE with no-admixture. The pie charts outside the map indicate the proportion of normal (blue) and androecium-mutant (red) flowers per population. The names of populations follow those given in Table 1.

The no-admixture analysis of STRUCTURE with correlated allele frequencies between populations obtained optimal likelihood scores for K = 6 to K = 10, whereas the admixture analysis with correlated allele frequencies inferred an optimal value of K = 9. The minimal and optimal no-admixture hypothesis (K = 6) found individuals of eight introduced populations belonging to just one genetic cluster (Red cluster: Bl/Il/Zi; Green cluster: Tl/Hw; Blue cluster: Tb/Pl; Pink cluster: Re; abbreviations for population names, see Table 1), individuals from eleven populations to belong to two different genetic clusters (Red/Pink: U/Wi/Gu/Bg; Red/Turquoise: Kt/We/A; Red/Green: Mu/Mt and Pink/Blue: Tp/Bz; abbreviations for population names, see Table 1) and individuals from one population to belong to three genetic clusters (Red/Pink/Blue: Gß; Figure 3; Genetic clusters are indicated by color, with capitalized color names). The three native populations belonged to two different genetic clusters (Turquoise cluster: Kz9 and Yellow cluster: UzT/Bk10). The most widespread genetic clusters were the Red and Pink clusters found within thirteen and eight populations along a distance of 130 km from Kirchentellinsfurt to Augsburg and from Tübingen to Großaitingen, respectively (Figure 3). Rare AFLP-fragments (with allelic frequencies below 0.15) represent 89% of the native area-specific fragments (40 of 45 AFLP-fragments) while they represent only 18% of the fragments common to both areas (22 of 121 AFLP-fragments). Moreover, fifteen of the 22 rare fragments common to both areas have a lower frequency within the introduced populations relative to the native populations (68%; data not shown).

**Table 1:** **Population genetic indices for the populations of *V. filiformis***

		**Code**	**N**_**samples**_	**N**_**clones**_	**Frag**. **polym**.	**%Frag. polym.**	**Frag. fixed**	**Frag. priv.**	**Hj**	**S. D.**	**Var.**
**All**		N= 23	108		261	88,78%	4	---	0.08023	0.03449	0.001190
**Native area**		N= 3	15		158	53,74%	8	46	0.13702	0.02739	0.000750
Locality	Region										
Uzungöl-Trabzon	*Turkey-Eastern*	UzT	4	0	72	24,49%	35	8	0.12754	0.01326	0.000176
Kazbegi-Kobi	*Georgia-Northern*	Kz9	5	0	97	32,99%	11	17	0.16790	0.01460	0.000213
Bakuriani-Tskratskaro	*Georgia-Central*	Bk10	6*	0	80	27,21%	42	13	0.11564	0.01154	0.000133
**Introduced area**		N= 20	93		213	72,45%	5	98	0.07171	0.02670	0.000713
Locality	Crossing group										
Tübingen	*blue*	Tp	3	2	24	8,16%	61	1	0.04488	0.00923	0.000085
Tübingen	*blue*	Tb	9	4	60	20,41%	45	3 (1)	0.07510	0.00935	0.000087
Tübingen	*red*	Tl	3	0	33	11,22%	70	0	0.06377	0.01087	0.000118
Kirchentellinsfurt	*green*	Kt	3	0	62	21,09%	26	3	0.12550	0.01419	0.000201
Pliezhausen	*blue*	Pl	7	6	52	17,69%	51	2	0.06997	0.00944	0.000089
Betzingen	*green*	Bz	4*	4	24	8,16%	69	3 (1)	0.05867	0.00959	0.000092
Reutlingen	*green*	Re	5	5	20	6,80%	71	1	0.03476	0.00697	0.000049
Hohenwittlingen	*green*	Hw	5***	5	31	10,54%	75	0	0.06321	0.01005	0.000101
Münsingen	*pink*	Mü	3	0	34	11,56%	52	1	0.06200	0.01125	0.000126
Mehrstetten	*violet*	Mt	3	0	27	9,18%	66	3	0.05312	0.00998	0.000100
Blaubeuren	*green*	Bl	4*	3	34	11,56%	55	6	0.06675	0.01022	0.000104
Ulm	*green*	U	5	3	43	14,63%	47	0	0.06364	0.00931	0.000087
Wiblingen	*green*	Wi	10*	9	45	15,31%	45	0	0.05465	0.00807	0.000065
Illerzell	*green*	Il	4	2	39	13,27%	45	3 (1)	0.06526	0.00982	0.000096
Günzburg	*green*	Gü	4	1	48	16,33%	42	3	0.07981	0.01078	0.000116
Burgau	*green*	Bg	4*	2	32	10,88%	63	2	0.05919	0.00983	0.000097
Ziemetshausen	*green*	Zi	3	3	37	12,59%	42	3	0.06627	0.01157	0.000134
Großaitingen	*unknown*	Gß	6	4	49	16,67%	47	6	0.06514	0.00895	0.000080
Westheim	*green*	We	4	0	75	25,51%	23	6	0.13030	0.01316	0.000173
Augsburg	*green*	A	4	0	73	24,83%	32	7	0.13221	0.01445	0.000209
	*all green*	13	59		185	62,93%	7	47	0.07694	0.03151	0.000993
	*all blue*	3	19		87	29,59%	35	7	0.06332	0.01617	0.000261
	*other*	4	15		102	34,69%	30	10	0.07171	0.02670	0.000713

n _pop._ = number of populations per area; Code = code of the population (see Table 1); N _samples_ = number of DNA-samples per population; the number of stars corresponds to the number of re-extracted DNA samples in the respective population used for error rate estimation; N _clones_ = Number of individuals belonging to a clone sampled multiple times; Frag. polym. = number of polymorphic AFLP-fragments; % Frag. polym. = percentage of polymorphic AFLP-fragments; Frag. fixed = number of fixed AFLP-fragments; Frag. priv. = number of private AFLP-fragments (number of private & fixed AFLP-fragments); S.D. = standard deviation; Var. = variance.

#### Clonality

Our AFLP primer combinations generated 294 unambiguous DNA-fragments from which 261 are polymorphic (88.78%). Four of them are fixed in *V. filiformis* and present in all DNA-samples (Table 1). The nine re-extracted individuals corresponding to our replicates gave an error rate of 4.65% (equal to 123 errors divided by nine pairs of replicates and 294 loci) according to Bonin *et al.*[56]. DNA-specimens which had a percentage of difference between their AFLP-fragments or a rate of pairwise individual comparisons below the error rate were considered as clones in this study. After two-by-two comparisons, 54 DNA-samples from 14 populations corresponded to 10 different clones (Figure 3 & Additional file 1): nine clones were population-specific (c2 to c10) whereas one clone was present within eight different populations (c1). This widely distributed clone represented almost all DNA-samples (N = 26 on 28) categorized by STRUCTURE as members of the Pink genetic cluster (Figure 3). Within our sampling, two populations were composed entirely of a single clone (c1 in Reutlingen (Re) and c8 in Ziemetshausen (Zi); Figure 3). In total, clonality was inferred for 58.06% of the DNA-samples from the introduced area (54 of 93 introduced specimens or DNA-samples) and 0.00% of the DNA-samples from the native region (0 of 15 native specimens).

#### Principle Coordinates Analysis (PCoA)

**Figure 4:**
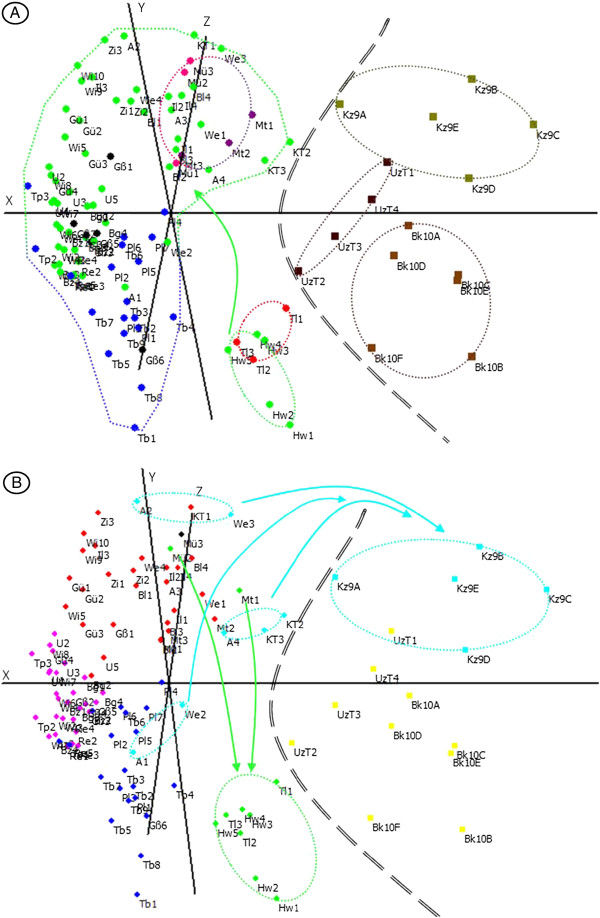
**Principle coordinate analysis of *V.filiformis *using Nei&Li**[57]**similarity distances.A**. Discrimination based on geographic region and crossing group. **B**. Discrimination based on genetic cluster. Colors for native populations correspond to the geographic distribution in Figure **A** and to genetic clusters identified during the STRUCTURE analyses of the AFLP experiment in part **B**. Samples left of the double dashed line are introduced populations, whereas those to the right are native populations. Colors for introduced populations correspond to crossing groups identified during the crossing experiments in part **A** and to genetic clusters identified in the STRUCTURE analyses of the AFLP experiment in part **B**. Black circles are individuals without crossing data from the Großaitingen population in part **A** and unclassified sample from the STRUCTURE analyses of the AFLP experiment (Mü) in part **B**. The dotted line corresponds to separate clusters highly supported (see Results for details). Hw = Hohenwittlingen; Tl = Tübingen Lustnau; Mü = Münsingen; Mt = Mehrstetten.

The PCoA based on standard similarity distances allowed the visual differentiation of clusters in our data set in three axes (x, y, z) accounting for 28.80% of the molecular variability (12.92%, 9.34%, 6.54%; Figure 4). Based on the PCoA coordinates, the native populations were separated easily from the introduced ones (Mann–Whitney-*U*-test: U = 28.8, p-value < 0.0001; Figure 4A). Within the introduced area, the groups of individuals based on the crossing experiment results (Figure 4A) can be compared with the genetic clusters of the STRUCTURE analysis in Figure 4B. Populations Mü and Mt belonged to different crossing groups (pink and violet groups, respectively, Figure 2) but cannot be distinguished from each other in the AFLP-analysis (p-value > 0.05; Figure 4A). The same is true for the samples belonging to the blue and green crossing groups (p-value > 0.05; Figure 4A). On the other hand, the individuals of the Hohenwittlingen (Hw) population are significantly distant to the remaining individuals belonging to the green crossing group (U = 10.84, p-value < 0.001; Figure 4A). Moreover, the samples of the Gß population belonged to three different genetic clusters (Pink, Red and Blue; Figure 4B compared to Figure 4A) but cannot be statistically differentiated (Red/Pink: U = 7.35, p-value = 0.0067 but with Red/Blue: U = 2.53, p-value =0.1110; Pink/Blue: U = 0.011, p-value = 0.917;Figure 4B).

#### AMOVA and genetic diversity

Native populations have higher genetic diversity Hj (0.137) than introduced populations (0.0727; Wilcoxon-Mann–Whitney-test: U = 68, p-value = 0.018; Table 1) and twice the number of polymorphisms (28.23% versus 14.32%; p-value = 0.002; Table 1). In contrast, introduced populations possess more fixed fragments than native populations (51 versus 29; p-value = 0.002; Table 1). Within the introduced populations, four introduced populations do not have any private fragment (Tl, Hw,U, Wi) while A has the highest number of such fragments (N_priv.frag._ = 7). The AMOVA-analysis found two-thirds of the genetic variation to be within areas (67.35%; native and introduced) and only one-third of the variation explained by differentiation between them (32.64%; Table 2). The six genetic clusters from the STRUCTURE analysis explain approximately the same differentiation (within genetic clusters 70.86% vs. among 26.75% genetic clusters, data not shown). Within the introduced area a small difference in the repartition of the genetic variation is observed between our two different groupings: crossing groups or genetic clusters. There is more genetic variation within the five crossing groups (81.84%) vs. among them (18.16%), and there is also more genetic variation within the five genetic clusters in the introduced populations (72.51%) than among them (27.49%; Table 2). The genetic variation explained by within-population variation is higher in the native area (74.52%) than within the introduced German populations (54.53%; Table 2). Within the introduced populations, a strong positive correlation exists between their genetic distances and their geographic distances (r = 0.293; p-value < 0.001; Additional file 2).

**Table 2:** Analyses of molecular variance (AMOVA) from AFLP fingerprints

**Sampling**	**Grouping (code)**	**N**	**Source of variation**	**d.f.**	**SS**	**Variance components**	**Percentage of variance**	**φ**_**ST**_
**All**	***Area***	2	Among areas	1	1.15	0.041	32.64%	0.326
	*(Nat./Intr.)*		Within areas	106	9.05	0.085	67.35%	
					V_total_ =	0.126		
**Native area**	***Region***	2	Among regions	1	0.25	0.015	9.27%	0.093
	*(Anat./G+L Cauc.)*		Within regions	13	2.28	0.156		90.73%
					V_total_ =	0.172		
	***Population***	3	Among populations	2	0.705	0.044	25.48%	0.255
	*(UzT/Kz9/Bk10)*		Within populations	12	1.575	0.131		74.52%
					V_total_ =	0.176		
**Introduced area**	***Population***	20	Among populations	19	3.779	0.034	45.47%	0.455
	*(20 transect pop.)*		Within populations	73	2.999	0.041	54.53%	
					V_total_ =	0.075		
	***Crossing group***	5	Among crossing groups	4	0.905	0.014	18.16%	0.182
	*(g/b/r/p/v)*		Within crossing groups	82	5.527	0.067	81.84%	
					V_total_ =	0.082		
	***Genetic group***	6	Among genetic groups	5	1.527	0.021	26.09%	0.261
	*(P/T/B/Y/G/R)*	Within genetic groups	93	5.252	0.059	73.90%		
					V_total_ =	0.080		

Nat. = native area; Intr. = Introduced area; Anat. = Anatolian –Pontic mountains (Turkish population); G+L Cauc. = Greater and Lesser Caucasian mountains (Kazbegi and Bakuriani populations); g/b/r/p/v = green, blue, red, pink and violet crossing groups, respectively; P/T/B/Y/G/R = Pink, Turquoise, Blue, Yellow, Green and Red genetic clusters, respectively; N = number of groups; d.f. = degree of freedom; SS = sum of squares; V_total_ = variance total.

### Androecium-mutant flowers

Among the ten investigated populations of *V. filiformis* in Germany (eight from the transect and one each in Bonn and Mainz; Figure 3), population Il had the highest percentage of mutant flowers (47 in 103 observed flowers; 45.63%), including 36 flowers that lacked filaments (Additional file 3C2), nine that had only one stamen (Additional file 3C3), one flower that had two stamens of different sizes (Additional file 3C1), and the unique case observed of one flower that had three stamens (Additional file 3C5). The Mt population had the lowest percentage of mutant flowers with 5.56% from a total of 54 counted flowers (Figure 3). Among the seven native populations investigated, only one had androecium-mutant flowers at low frequency (1.48%; Kz8). The populations of native *V. chamaedrys* (1.85%; N_flower_ = 379) or introduced *V. persica* (0.20%; N_flower_ = 507) from Mainz present a markedly lower percentage of mutants than the neighboring population of introduced *V. filiformis* (15.19%; N_flower_ = 953) and any other introduced populations but not the native populations. Significant differences exist between the native and introduced populations (p=0.01).

### Pollen and ovule production

Number of pollen grains per flower differed significantly between areas (flowers in the native area had more pollen grains; p<0.001), crossing groups (p=0.038) and populations (Additional files 4, 5). Within the native area the population from Cross Pass (Kz8) produced significantly less pollen (p<0.001) than the other native populations. Ovule numbers differ significantly only between crossing groups (p<0.001; Additional files 4, 5) with green and violet crossing groups producing more ovules than the rest. Plants from the native area appear to produce less ovules, but this is not significant (p=0.130).

### Seed production

**Figure 5:**
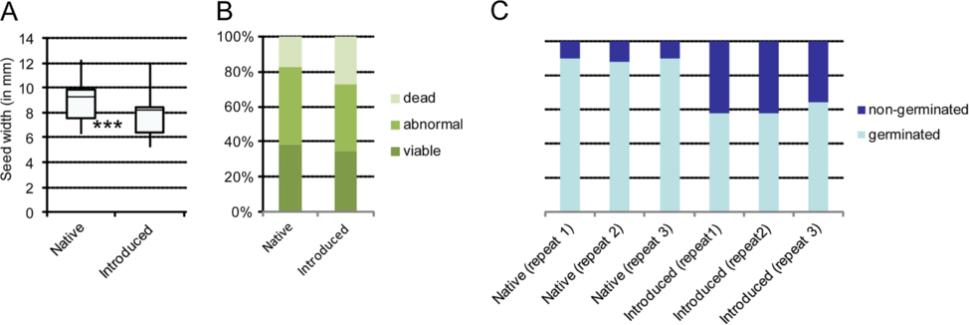
**Seed variation between seeds collected in the native area and those produced from German plants of *V.filiformis. *A**. Seed size (width). **B**. Seed viability test. **C**. Seed germination test. Significance level: * = P ≤ 0.05, ** = P ≤ 0.01 and *** = P ≤ 0.001; 1 graduation = 0.12 mm.

Capsules produced in the greenhouse after hand-pollination contained more seeds than capsules collected in the field after open-pollination (Kruskal-Wallis-test: H = 5.064; p-value = 0.024; Additional file 6). Variation in seed number per capsule was also found between the native and introduced area and within the introduced area between populations, crossing groups and genetic clusters (Additional file 6). Seeds from the introduced area were also smaller than seeds of the native area (Figure 5A; Kruskal-Wallis-test: H = 88.053; p-value < 0.001). Significant differences were found between Georgian populations for seed size (H = 169.202; p-value < 0.001; Additional file 6). In the REML analysis for seed number, area and population were the most important factors as indicated by AIC and BIC (699.66, 719.61 respectively), which means that the number of seeds per capsule is different between areas and between populations. For seed size using the mean width of seeds per capsule, the model with area and number of seeds per fruit as fixed factors and population as random factor proved to be the best (AIC −172.69; BIC −156.51). The next best model had an AIC difference of 2 and included more parameters, and was thus discarded [58]. For the introduced area alone, again, population had the strongest effect (AIC −60.99; BIC −50.61), whereas crossing group and genetic group were less important. The model was again better for the above mentioned criteria. For seed number the model with area and population was also the best (AIC 699.66; BIC 719.61).

For seed viability, a significantly lower number of dead seeds was found in the native versus the introduced plants (H = 1.84; p-value = 0.033; Figure 5B). Within the Tl-Pl-Re triangle of the introduced area, seeds produced by the widespread green crossing group and the Pink genetic cluster (Figure 3) have a significant lower viability (22% viable seeds) than the seeds produced by the two other crossing groups or genetic clusters (red crossing group and Green genetic cluster: 44% viable seeds, t = −30.98; P-value = 0.001 and blue crossing group and Blue genetic cluster : 36% viable, U = −3.24; p-value = 0.042) irrespective of the pollen donor (see below). A maternal effect was also found for seed size with plants belonging either to the blue crossing group or the Blue genetic cluster producing significantly bigger seeds than the ones from the other crossing groups or genetic clusters (data not shown). No paternal effect was found by comparing the results of seed viability and size between the same combinations ♀ - ♂: Tl-Pl/Pl-Tl; Tl-Re/Re-Tl and Pl-Re/Re-Pl (data not shown). Moreover, the seeds from the native area germinated more frequently than the ones from introduced populations during the three repeats (90% vs. 58%, s.d. 1.15) and this difference in germination rate is statistically significant (H = 3.97; P-value = 0.046; Figure 5C).

### Correlations between data sets

Positive correlations were observed between pollen production per flower and a) the number of polymorphic AFLP-fragments (r = 0.695; p-value = 0.019; Additional file 7C1), b) Nei’s gene diversity (Hj; r = 0.722; p-value = 0.019, data not shown) and c) private AFLP-fragments per population (r = 0.612; p-value = 0.040; data not shown). Negative correlations were found between pollen production and the number of fixed AFLP-fragment (r = − 0.793; p-value = 0.005; Additional file 7C2) and the percentage of clones per population (r = − 0.744; p-value = 0.011; Additional file 7D1). Negative correlations exist between the percentage of androecium-mutant flowers and a) pollen production per flower (r = − 0.508; p-value = 0.046; Additional file 7A and with b) number of polymorphic AFLP-fragments per population (r = − 0.579; p-value = 0.040; Additional file 7E1) and c) number of private AFLP-fragments per population (r = − 0.592; p-value = 0.036; data not shown). Conversely, ovule production is not correlated with flower mutation frequency (Additional file 7B) or with population genetic data (data not shown). It should be mentioned that the genetic diversity (Hj) is not correlated with the number of DNA-samples or specimens investigated per population (r = − 0.067; p-value = 0.380), but is instead significantly and positively correlated to the proportion of non-clonal specimens per population (N _no clone_/N _DNA sample_; r = 0.765; p-value < 0.001; Table 1), and negatively correlated with the proportion of clones per population (r = − 0.800; p-value < 0.001).

## Discussion

### **Invasion without seed**: **Is V**. **filiformis sterile in Europe**?

Ever since the first record of *V. filiformis* was documented growing around the roots of Georgian vine shoots near Marseille (Aubagne, southern France, 1893; [39]), European populations of this species have been considered to be sterile by botanists because no seed set has been observed in the introduced area [39,45,48]. Bangerter & Kent [42] reported three plants with capsules out of more than 300 specimens checked, and Lehmann [39] summarized similar observations from other botanists.However, capsule formation alone is not proof of sexual reproduction. In our crossing experiments, capsules formed occasionally but did not necessarily contain seeds, an observation previously made by Lehmann [39]. In the course of our study, and despite our intensive investigations, we have neither observed capsules in the field nor in herbaria. A report of seed set in *V. filiformis* in Switzerland [59] has been shown by us to be due to misidentification (pers. obs.). Based on this observation and previous unsuccessful crossings by Lehmann [40] in one population from Tübingen Botanical Garden and Thaler [60] in three populations from Graz, we decided to limit our intrapopulation crossing to a single, population that contained many flowering individuals. This population likewise did not produce seeds. Subsequent investigations in populations from Oldenburg further support this view (Marszalkowska, unpubl.).

Two other aspects make sexual reproduction in the introduced populations of *V. filiformis* improbable. First, pollinators observed by Thaler [46] in introduced populations are small Diptera and Hymenoptera, which are territorial and rarely fly more than one meter [61,62]. Thus, long distance pollinator movement between clones with different S-alleles or between populations is unlikely. Even in the three geographically close populations (Tl-Pl-Re, minimum distance four kilometres), which were demonstrated to produce seeds in the inter-population crossing experiments (Figure 2), no seed production was found in the field. Second, the majority of introduced populations are located in public or private gardens, turfs or parks, which are mowed during the flowering period [54]. Consequently, even rarely produced fruits would be removed prior to seed maturation, which occurs at least one month after successful pollination (pers. obs.).

Although the absence of seed set after intrapopulation crossings could be explained by general sterility, the ability to set seed successfully was demonstrated by our interpopulation crossing experiments in the greenhouse. This means that the reproductive system of European *V. filiformis* is still functional (Figure 2). Consequently, the invasion process was not facilitated by the breakdown of the SI system or the disruption of the sexual reproduction as found in other species [10,63-65]. Instead, clonal reproduction either has substituted sexual reproduction, or is at least prolonging the phase until sexual reproduction can occur again, leaving the possibility of future sexual reproduction.

### **Does the population structure of V**. **filiformis differ between the introduced and native range**?

In the absence of sexual reproduction, *V. filiformis* relies on vegetative reproduction for colonization of a new area. In our study, AFLP-fingerprinting data revealed widespread clonality in the introduced area. Our genetic analyses show that clones are common in the introduced populations and indeed there are even several monoclonal populations (Figure 3 & Additional file 1), whereas no clonal individuals have been found in populations of the native area. Similar results, where clonal reproduction is restricted to introduced populations, have been found in other invasive weeds such as *Rubus alceifolius*[66] or *Fallopia japonica*[14]. The presence of clones in *V. filiformis* is also verified by the absence of a relationship between the number of genetically investigated individuals per population and the level of intra-population genetic diversity. However, such a correlation appears when clonal samples are deleted from the analysis (data not shown). Moreover, the significant difference between the distribution of molecular variance among and within native vs. introduced populations and the difference in the fixation index (φ_ST_; Table 2) support the hypothesis of a difference in population structure, with predominant to exclusive clonal reproduction in the introduced area. Indeed, the molecular variance is distributed equally among and within the native populations (among: 25.48%/within: 74.52%) as well as among and within the genetic clusters of the introduced area (among: 26.09%/within: 73.90%) compared with that among and within the introduced populations (among: 45.47%/within: 54.53%; Table 2). Although we have identified extensive clonal reproduction, intra-population genetic diversity within introduced population is not zero (Table 2) and may be due to either i) the accumulation of somatic mutations within clones as is the case for the self-incompatible and asexually reproducing *Oxalis pes-caprae*[36] and the single clone of dioecious *Sphagnum palustre* on Hawaii [67], or ii) the presence of several clones of multiple introductions, which either share the same S-alleles or do not produce seeds for other reasons (Figure 3). At present we are not able to distinguish between these. Comparing the genetic diversity in the populations from different parts of the native range with that in the introduced populations (Figure 4) it is obvious that a substantial amount of genetic diversity has been introduced.

### **How many introductions of V**. **filiformis have occurred**?

It is tempting to hypothesize that our five genetic clusters identified by the assignment tests correspond to five different introductions, but the situation is certainly more complex. Our AFLP-fingerprint data suggest that two genetic clusters (Red cluster, thirteen populations between Kt and A/Pink cluster, eight populations between Tp and Gß) have been dispersed 130 km across the study area (Figure 3). These two genetic clusters could easily correspond to separate introductions, but this is likely to be an underestimate for the number of separate introductions, as separate introductions of closely related plants cannot be distinguished. The large zone covered by individuals belonging to the same genetic cluster in this non-native environment is accompanied by low frequency or absence of private fragments (Table 1). The successive introductions during the colonization of southern Germany must have produced a succession of founder effects or bottlenecks as observed in *Fallopia sp.*[14] or *Aster furcatus*[65]. However, we were not able to distinguish subsequent bottlenecks from the initial introduction bottleneck, indicated by fewer polymorphisms, lower intrapopulation genetic diversities (Table 1) and by a reduction of the frequency of rare fragments (f < 0.15; data not shown) in introduced populations compared to the native ones. Since specimens belonging to the Red, Blue and Pink genetic clusters cannot be significantly discriminated in the PCoA analysis, they are inferred to be closely related. This suggests that these genotypes came from a single region, as previously implied by Lehmann [39] based on the information on plant traders that introduced the species. In the extreme case, it is even possible that they correspond to a single introduction and fixation of different S-alleles and subsequent somatic mutations in different populations.

Several incongruencies exist between crossing and genetic data, for example: i) populations Tp and Bz are assigned to the same genetic clusters (individuals either in Blue or Pink clusters) but are inferred to contain individuals with different S-alleles (blue and green crossing groups; Figure 3); ii) individuals in Mü and Mt are also in the same genetic clusters (Red and Green clusters) but are again inferred to have different S-alleles (pink and violet crossing groups; Figure 3) and none is the green one. Such divergent S-alleles could either be introduced or evolved post-introduction. Alternatively, some post-introduction sexual reproduction could have allowed genetic exchange, especially at the S-locus, but the small population size would have led to a gradual fixation of two S-alleles in a particular population (S-Allee effect; [68,69]).

The opposite case, genetically divergent individuals in the same crossing group, can be explained by the same S-allele being derived from a genetically differentiated introduction and fixed in different populations. One case may be the Pink and Red genetic clusters, which largely correspond to the green crossing group (Figure 3). Another example may be the population Hw, which is incompatible to other green populations although it is alone with the Tl population in the Green genetic clusters (Figure 3).

Thus, between three and five genetically distinct introductions can be inferred within the introduced area but the actual number cannot be estimated. The correlation found between geographic and genetic distances (Additional file 2) is characteristic for multiple introductions as already observed by others [30,66]. Moreover, three introduced populations (Kt, We and A), which are 120 kilometres apart from each other and have the highest genetic diversity of the German populations, are grouped together with the native population located in the Greater Caucasus (Kazbegi-Kobi) in the same genetic cluster (Turquoise cluster; Figure 3 and Table 1). This indicates a possible north-eastern Georgian origin of these German populations, as opposed to the two other native regions (north-eastern Turkey and central Georgia) as putative sources. However, detecting precisely the sources of each introduced population would require a phylogeographic analysis with a denser sampling in the native distribution area.

### **Consequences of an absence of sexual reproduction**: **Muller**’**s ratchet in action**?

Since sexual reproduction no longer occurs in the introduced area, selection pressure on primary sexual floral characters has ceased compared to the native area. Thus, mutations affecting the reproductive parts fail to have a serious fitness effect and are free to accumulate [12]. This accumulation of deleterious mutations without the chance to reduce them again in organisms without meiotic recombination has been termed Muller’s ratchet [70,71], and has been shown to lead to an increased extinction risk in asexual organisms [72]. *Veronica filiformis* is able to tolerate deleterious mutations affecting reproductive organs (in the androecium) better than other species since it can reproduce clonally in an effective manner. Nevertheless, mutations affecting the reproductive parts are considered deleterious since the lack of recombination prevents the reduction in mutation load. Mutations in flowers within introduced populations can be observed frequently (between 5% and 45% of flowers are affected within fifteen populations investigated; Figure 3) compared to their rare occurrence in the native range (~1.5% within one of seven populations). Similarly, they are much less frequent in other species of the genus [44], including the self-compatible introduced *Veronica persica* (0.20%) and the self-incompatible native *Veronica chamaedrys* (1.85%) studied here, which are both able to reproduce clonally but not exclusively so [73,74].

Our investigations indicate that the androecium-mutations in introduced populations of *V. filiformis* also affect pollen production. First, introduced populations produce a highly significantly lower number of pollen grains (30% less; Additional file 4). Second, the single native Georgian population possessing androecium-mutant flowers (Cross Pass Kz8) has a significant lower pollen production than the other native populations (40% less; Additional file 4). Third, a negative correlation was found between the percentage of androecium-mutant flowers counted per population and the pollen produced by these populations (r = − 0.5076). Since we assumed an origin of the German populations from the Greater Caucasus, where the single native population with androecium-mutant flowers (Kz8) was found, it is possible that the genetic basis for the androecium mutations was inherited from the source area. However, another possibility is that this population itself has a history of extensive vegetative reproduction with relaxed selection on floral reproductive characters since a partial alteration of the environment has been observed on the site (pers. obs.).

Although we observed mutations only in the male reproductive parts (stamen, pollen) in *V. filiformis*, female traits involved in sexual reproduction should be affected similarly and were inferred by the observed maternal effect on seed size and seed viability. Unfortunately, we were unable to bring living plants from the native area to the greenhouse and, therefore, we were only able to compare seeds produced in the greenhouse from introduced populations to those produced in the field in the native area. Differences between seed characters produced in the field versus greenhouse have commonly been observed, with seed germination rate being lower [75] and seed size being smaller in field-reared seeds [76], likely due to better nutrient supply and less competition in the greenhouse. Thus, a seed number-seed size trade-off, for example, could be invoked to explain this difference, based on differences in ovule production between genotypes (Additional file 4) or pollen limitation in the native habitat as indicated by fewer seeds per capsule in native plants compared to those from capsules produced in our artificial crossings. However, we demonstrated that even when we controlled for seed number by estimating average seed size per capsule, seed size still differs between areas, which also explains why our observed differences in germination rate and seed size (Figure 5) are opposite to those expected (see above). Furthermore, the detected differences in seed viability between introduced populations of different crossing groups and different genetic groups on seed viability demonstrate that genetic factors influencing seed viability exist even within the introduced area. Finally, differences in seed size and germination rate parallel those observed in embryo size (data not shown) and number of dead seeds (Figure 5B; without a difference in ovule-seed ratio) between seeds of the two areas. Therefore, we consider the best explanation for these phenotypic mutations to be the genetic consequences of deleterious mutations in the genome accumulated by Muller’s ratchet, which occurred after the cessation of sexual reproduction and affects various life stages.

Similar to our observations, a relationship between pollen viability and other morphological characters and the age of the population were detected previously in other species that reproduce vegetatively [77,78]. The observed relationship between age of meadows, pollen viability and petal number in those studies suggests a similar effect of accumulation and finally fixation of mutations deleterious to sexual reproduction. Unfortunately, for most of the populations in our study, the time of colonization is not known and thus the gradual accumulation of mutations suggested by Muller’s ratchet cannot be inferred directly. However, the analysis of correlations between the proportion of androecium-mutant flowers, pollen production and genetic diversity offers some indirect evidence for the gradual accumulation of mutations. The results of correlations taken together (Additional file 7) suggest that the more clonal the populations and the lower the number of polymorphic AFLP fragments, the lower the number of flowers per individual, the lower the pollen quantity, the higher the frequency of dead seeds per capsule, and the higher the frequency of mutant flowers. Whereas the underlying common cause for these observations is not demonstrated per se, the most likely explanation is accumulation of mutations affecting various life stages and impairing sexual reproduction. Thus, our preferred scenario for the colonization of new populations of *V. filiformis* involves the introduction of a few but genetically divergent individuals that first lost the ability to reproduce sexually because there were not enough compatible S-alleles in the populations (Allee effect). Subsequently, the lack of sexual reproduction led to an erosion of genetic diversity and increasing importance of clonal reproduction in the populations. This erosion of genetic diversity was paralleled by the accumulation of mutations leading to a decrease in quantity and viability of pollen and seeds, as well as a reduction in the number of flowers per individual and an increase in phenotypically discernible mutants. The predicted endpoint of this seems to be reached in Großaitingen (Gß), a population apparently lacking the ability to flower altogether.

## Conclusions

*Veronica filiformis* proved to be an excellent example to demonstrate the consequences of human introduction of a plant species to a new environment. This obligate self-incompatible species, which is native to the high mountains around the Black Sea, colonized Europe and North America after introduction by gardeners or plant traders at the start of the last century, apparently exclusively by vegetative means. Currently, there is no evidence for sexual reproduction in the introduced area based on field observation and intrapopulation crossing. Thus, clonal reproduction serves as a means of reproductive assurance. Therefore, the clonal diversity detected by DNA fingerprinting is the product of conserved diversity of introduced plants not corresponding to S-allele diversity and somatic mutations that occurred after the loss of S-allele diversity. In the future, selection of individuals for intrapopulation crossing should be guided by an analysis of genetic relationships to exclude any possibility that sexual reproduction in introduced populations is possible.

Spread of single clones of *V. filiformis* across more than 100 kilometers was likely facilitated by watering and mowing of the European turfs, but did not lead to the merging of compatible populations even when compatible clones are in populations less than four kilometres apart. Nevertheless, the potential for sexual reproduction still exists and neither genetic diversity nor clonal dispersal seem to be limiting factors. Comparing genetic diversity, population structure and pollinator guilds with that in the native area would be necessary to evaluate future potential for sexual reproduction in the introduced area.

The spread of *V. filiformis* to Europe came at an evolutionary price: the accumulation of mutations deleterious to sexual reproduction following Muller’s ratchet. *Veronica filiformis* is shown here to be one of a very few clear examples for this phenomenon, which is detectable in various parts of the flower (androecium, pollen, ovule, seed, embryo). The flower has thus lost its fundamental and evolutionary role for population maintenance in the species’ introduced range. Furthermore, there is some indication that mutation frequency increases with population age. It remains to be investigated whether this deterioration of the reproductive system is compensated by increased vegetative growth. The range of populations differing in the amount of mutations, from native populations to that in Großaitingen that apparently has lost the ability to produce flowers, offers an excellent model to investigate this trade-off and the genetic basis of Muller’s ratchet.

## Methods

### Plant material

Between March and April 2007, plants from eighteen populations of *Veronica filiformis* were collected along a transect between Tübingen (Baden-Württemberg), Ulm (Baden-Württemberg) and Augsburg (Bavaria) with a maximum distance of 150 km (T-U-A; Figure 1; Additional file 1). The sampling was guided by the first observations in Germany based on records by Lehmann [39] and Thaler [49] in Tübingen Lustnau (1909), old cemetery of Ulm at Wiblingen (1936), and unspecified public parks of Augsburg (1939). Since three populations (Tubingen Lustnau Tl – Pliezhausen Pl – Reutlingen Re) separated by distances betweenfour (minimum) and ten kilometers (maximum) showed seed production in crossing experiments of 2007, two supplementary populations (Kirchentellinsfurt Kt – Betzingen Bz) were collected within the triangle formed by these populations. At each locality at least ten putative individuals of *V. filiformis* (with a maximum distance between each other and without connection between them) were collected in plastic bags with wet paper tissue in order to keep them alive during the collecting trip. Afterwards, these plants were transplanted to a greenhouse of the botanical garden of the Johannes Gutenberg-University, Mainz, Germany. Field trips were taken to five regions of the native range of the species to collect seeds and silica-gel dried material, including northern Turkey (2005), and eastern (Kakheti, Lg), central (Samtskhe-Javakhéti, Bk), northern (Kazbegi, Kz) and south-western (Adjara, Bt) Georgia (2008) (Figure 1; Additional file 1). Georgia is the most likely source of origin for introduced populations [40] and northern Turkey represents a distinct different part of the distribution range. Voucher specimens are deposited at the herbarium of Staatliches Museum für Naturkunde Stuttgart (STU).

### Sampling and crossing experiments

Plants from each locality were covered collectively by tissue or plastic lids to prevent unintentional cross-pollination and each putative individual (10–12 per population) was potted and labeled separately. Fertilizers (10ml Hakaphos soft Spezial per pot; 16%N, 8% P_2_O_5_, 22% K_2_O, 3% MgO with <0.2% boron, copper, iron, manganese, molybdenum, zinc) were used at the start of the experiment once to improve the success of the transplantation. Flowers are slightly protogynous and the virginity of the stigma was checked by binocular microscopes before crossing. Typically one anther with mature white pollen was used only once to fertilize a single style using sterile forceps in order to deposit enough pollen per style and prevent other crossings. Labeled threads with a code were attached to the pedicels after crossing in order to identify the flowers later. The code, date of crossing, the father individual and its population together with the mother individual and its population were noted. Presence or absence of developing fruits was checked regularly after each crossing. To confirm the absence of capsule production within introduced populations, eight specimens from the population Pliezhausen Pl were crossed with each other (n _flowers_ = 41). In inter-population crosses, priority was given to crossings of neighboring populations and only surplus flowers were used to cross geographically distant populations to check if these belong to the same crossing group (Figure 2).

### DNA extraction and AFLP generation

A total of 108 DNA-samples considered to be putative individuals from twenty introduced and three native populations (Table 1) were sampled for AFLP fingerprints. DNA-samples do not correspond to the samples used for the crossing experiments although they come from the same German populations. Fresh leaves of the individuals were dried in silica gel and used to extract total genomic DNA by DNeasy^TM^ plant minikit (Qiagen) following the manufacturer’s instructions. DNA concentration was measured spectrophotometrically with a GeneQuant RNA/DNA calculator (Pharmacia, Uppsala, Sweden), or estimated visually by ethidium bromide staining on agarose gels. The AFLP^TM^ procedure followed Vos *et al.*[79] with the following modifications. Genomic DNA was digested with the two restriction endonucleases EcoRI and MseI and ligated to double-stranded EcoRI (5’ – CTCGTAGACTGCGTACC – 3’; 3’ – AATTGGTACGCAGTC - 5’) and MseI (5’ – GACGATGAGTCCTGAG – 3’; 3’ – TACTCAGGACTCAT - 5’) adapters in one step at 37°C for 3 hours. The reaction mix for circa 0.5 μg template DNA contained 1.1 μl T4 DNA ligase buffer (Genecraft, Cologne, Germany), 1.1 μl 0.5 M NaCl, 0.55 μl bovin serum albumin (BSA, 1mg/ml, New England Biolabs, Beverly, Massachusetts), 1.0 μl 50 μM MseI-adapters (Metabion, Martinsried, Germany), 0.1μl MseI (10U/μl, New England Biolabs), 1.0 μl 5 μM EcoRI-adapters (Metabion), 0.25μl EcoRI (20U/μl, New England Biolabs), 0.002 μl T4 DNA ligase (10WU/μl, Genecraft) and 0.9 μl ddH2O. Ligated DNA fragments were diluted 10-fold. Preselective and selective amplifications were performed in a thermocycler (GeneAmp PCR System 9700, Applied Biosystems, Foster City, California) with PCR protocols following Vos *et al.*[79] with modifications. The reaction mix for preselective amplification contained 2.5 μl 10× NebTaq PCR reaction buffer (New England Biolabs), 0.1 μl NebTaq PCR reaction mix (5U/μl, New England Biolabs), 0.25 μl 10 mM dNTPs (Applied Biosystems), 0.50 μl 5 μM preselective primers E01 (5’ – GACTGCGTACCAATTCA – 3’) and M02 (5’ – GATGAGTCCTGAGTAAC – 3’), 6.65 μl ddH2O and 2 μl diluted product of the restriction-ligation reaction. The PCR product was diluted 10-fold. The reaction mix for the selective amplification contained 1.667 μl 10× NebTaq PCR reaction buffer (New England Biolabs), 0.055 μl NebTaq PCR reaction mix (5U/μl, New England Biolabs), 0.167 μl 20 mM dNTPs (Applied Biosystems), 0.833 μl 50 mM MgCl2, 0.28 μl 5 μM MseI-primer (Metabion), 0.20 μl 1 μM EcoRI-primer (Metabion), 7.92 μl ddH2O and 5 μl diluted product of the preselective amplification. The six primer combinations for the selective PCR were E38 - Hex (5’ Hex - GACTGCGTACCAATTCACT - 3’) combined with M56 (5’ - GATGAGTCCTGAGTAACGC - 3’); E38 - 6 - Fam/M50 (5’ - GATGAGTCCTGAGTAACAT - 3’) and E37 - Ned (5’ Ned - GACTGCGTACCAATTCACG - 3’)/M54 (5’ - GATGAGTCCTGAGTAACCT - 3’); E38 - Hex combined with M62 (5’ - GATGAGTCCTGAGTAACTT - 3’); E38 - 6 - Fam/M53 (5’ - GATGAGTCCTGAGTAACCG - 3’) and E37 - Ned/M51 (5’ - GATGAGTCCTGAGTAACCA - 3’). 2.5 μl of 6-Fam - 3.75 μl of Hex and 3.75 μl of Ned labeled products of each sample were combined, and 2 μl of this multiplex product was run with 7.75 μl HiDi formamide (Applied Biosystems) and 0.25 μl internal size standard GeneScan ROX (Applied Biosystems) on an ABI 3130x automated capillary sequencer. Raw AFLP data were collected and aligned with the internal size standard using ABI Prism GeneScan analysis software 3.7 (Applied Biosystems). Peaks (i.e. fragments) were scored manually as present (1) or absent (0) in a readable region of bands from 75 to 500 bp in length with GeneMarker version 1.5 (GeneMarker, SoftGenetics, State College, Pennsylvania). Each peak with a signal intensity of more than 1000 was selected and checked for presence in each sample.

### AFLP data analyses

First, the mismatch error rate based on nine (8.3%) supplementary re-extracted samples (or replicates) belonging to one native and six introduced populations was calculated to evaluate the quality of our analyses and determine the clonality threshold as the number of genotype mismatches divided by the number of replicate pairs and the number of loci [56,80] Table 1. The presence/absence matrix generated with the six primer combinations was imported in FAMD v. 1.1 [81] to assess genetic diversity between areas, regions, populations, crossing groups or genetic clusters. The genetic diversity is represented, within these different levels of analysis, by the mean of AFLP fragments present per individual, the number of polymorphic fragments (Frag. polym.) and its percentage (% Frag. polym.), the number of fixed fragment (Frag. fix.), the number of private fragment (Frag. priv.), the number of private and fixed fragments, and finally Nei’s gene diversity (Hj). Analyses of molecular variance (AMOVA) were conducted using the same software. To estimate if differences exist between samples (area, population, crossing groups and genetic clusters) within the genetic data, the non-parametrical Wilcoxon-Mann–Whitney-test (W/U) for two groups or the Kruskall-Wallis-test (H) for more than two groups were conducted using SPSS v 15.0 [82]. Genetic structure of populations was analyzed using the software STRUCTURE vers. 2.3 [83]. First, most of the parameters were set up as recommended in the user’s manual of STRUCTURE [83] and then, the option of “correlated allele frequencies” between populations was tested as recommended by Falush *et al.*[84] in case of subtle population structure. From a pilot study, we found that a burn-in period and MCMC (Markov chain Monte-Carlo) of 10000 and 50000 iterations, respectively, were sufficient. A series of 10 runs was conducted for each value of K populations between 2 to 23 (maximum number of localities) and the maximum value of Ln (Prob) obtained for each K was used to represent the best population structure acquired with the no-admixture and admixture models. The no-admixture model was chosen since the amount of admixture was found to be negligible. A Principle Coordinates Analysis (PCoA) was performed using Nei&Li’s coefficient [57] and the coordinates of each individual were extracted to statistically test their distributions by the same Wilcoxon-Mann–Whitney and Kruskall-Wallis tests using SPSS v. 15.0 [82].

### Flower characters

In the course of our crossing experiments, normal flowers (Additional file 3A &2B) and flowers with different types of mutations in the androecium (Additional file 3C1-5) were observed in the greenhouse and in the field. These different types of androecium-mutant flowers were counted in ten introduced and seven native populations (Figure 3 and Additional file 1) in the field or greenhouse. To check if these flower mutations are specific to *V. filiformis* (*V.* subgenus *Pocilla*), these particular mutant flowers were also counted in populations of two other *Veronica* species: the closely related *V. persica* (invasive in Europe; self-compatible) and *V. chamaedrys* (native in Europe; self-incompatible), both of which were growing both spontaneously near one of the *V. filiformis* populations (arboretum of the botanical garden of Mainz; Mz). The number of mutations in the androecium were compared with a generalized linear model (glm) function glm in R [85]. The differences between native and introduced regions were tested with a glm for binomial data with area and populations as factors.

### Pollen and ovule characters

To estimate the investment in gametophytes, at least eight buds per population in the five native and eight introduced populations of *V. filiformis* were sampled for a total of 135 mature buds (Additional file 1). Mature buds were collected in the field or in the greenhouse of the botanical garden immediately prior to anthesis and preserved in 0.9 ml of 70% ethanol. Each mature bud came from one separate individual. To count pollen grain number, the two anthers of one flower were dissected carefully under the binocular microscope. Two drops of methylene blue MTT (3 g of methylene blue in 1 l of 20% ethanol) and glycerine were added to the suspension for a total of 1 ml and stirred together for 30 sec with a vortex mixer. Then, 1 μl was transferred onto a slide and pollen grains were counted with the aid of a microscope. This was repeated ten times per flower. The total number of pollen grains was calculated as the mean of the counted pollen grains in the ten drops multiplied by 1,000 (factor of dilution). Ovaries were carefully dissected and the number of ovules per flower was counted under the binocular microscope. The pollen/ovule ratio (P/O) was scored as the total number of pollen grains in the two anthers divided by the number of ovules in the ovary of one flower.

Both the pollen and ovule production were tested for normality and homogeneity of variance by checking the residuals as stated useful by Quinn & Keough [58]. A histogram plot of each parameter showed a normal distribution and also residuals did not increase with increasing parameter values. For both parameters a nested ANOVA was conducted in R [85] with the lm function. Area (introduced vs. native), crossing group and populations were used as factors.

### Seed characters

#### Seed number and size

After each crossing, maturity of capsules was monitored daily until capsule opening. Seeds were collected in paper bags and stored in a dry place. Number of seeds per capsule was noted and length and width of each seed were measured under a graduated microscope. To test for differences in the success of sexual reproduction, the number and size of seeds produced in the crossing experiments of introduced populations were compared with the number and size of seeds collected from open pollination in populations in three native regions in Georgia. This was done first by the non-parametrical Kruskall-Wallis-test (H) using SPSS v. 15 [82], and second by an REML based approach (using lmer() in R package lme4 [86]). Different models were tested for seed numbers and seed size to find the best explaining model according to the AIC and BIC criterion by comparing the different models in an ANOVA (using anova() in R [85]) and pairwise comparison of the full model to models with similar AIC/BIC values. For seed size (the mean width of the seeds per capsule) the models contained area and seeds per fruit as fixed factors and region and population within region as random factors. The different models were tested with an ANOVA. Additionally, models were tested for the introduced area with the factors crossing group and genetic group. For seed number, area was used as fixed factor and region and population as random factors. Number of seeds per fruit was treated as continuous in the R analyses.

### Seed viability

Viability of seeds from crossings was examined in the populations of the triangle Tl-Pl-Re. These populations belong to different crossing groups (red, blue and green crossing groups; Figure 1) and different genetic clusters (Green, Blue and Pink genetic clusters; Figure 2). These seeds were used to study maternal and paternal effects on seed size and viability within the introduced range. At least twenty seeds were tested in each combination of maternal and paternal parent (♀ - ♂: Tl-Pl, Pl-Tl, Tl-Re, Re-Tl, Pl-Re and Re-Pl) to have an equal number of seeds from the same mother and father population. Fifty seeds from three regions in Georgia (Adjara, Samtskhe-Javakhéti and Kakheti) were used to compare seed viability between introduced and native ranges. A total of 150 seeds from the introduced area were compared with 150 seeds from the native area by tetrazolium staining [87-89]. Seeds were sliced longitudinally through the midsection of the distal half, then placed in petri dishes with 2.5 ml of 1% 2,3,-triphenyl tetrazolium chloride. After staining for 24h, seeds that displayed a completely red-stained embryo and endosperm were classified to be “viable” [87-89]. Other distribution of color were considered to be “abnormal seeds” (i.e. seeds with mixtures of red and white in one tissue, likely staining artifacts of otherwise viable seeds) or “dead seeds” (i.e. seeds with one or both organs white). Seed viability was calculated as the number of viable seeds in the total of tested seeds for each area (introduced/native) or each mother ♀/father ♂ group (Tl/Pl/Re) and statistically tested by the non-parametrical Wilcoxon-Mann–Whitney-test with SPSS v. 15 [82].

### Seed germination

A seed germination test [90,91] was conducted and adapted to *V. filiformis*. 150 seeds from two of the three previously cited native Georgian regions (Samtskhe-Javakhéti and Kakheti) and 150 seeds from a random sample of cross-compatible introduced populations from inside and outside the German transect (Baden-Württemberg and Bavaria) were investigated in three batches of 100 seeds each. Seeds were stored at 5°C for two weeks before being placed in wet Petri dishes (with fungicide added). The number of germinated and non germinated seeds was counted after a temperature treatment of 30°C - 8 hours/15°C - 16 hours during a period of 28 days. For the same reason as for seed size, we pooled the seeds and compared results only by the general non-parametrical Wilcoxon-Mann–Whitney-test using SPSS v. 15 [82].

### Correlations

After observing the differences between introduced and native plants we wanted to test whether Muller’s ratchet acted as a response to lower genetic diversity and increased clonality of populations. Our initial hypothesis was therefore that the percentage of clonality was positively correlated with androecium mutants and fixed AFLP markers, but negatively correlated with number of polymorphic loci, gene diversity (Hj), number of private AFLP fragments, seed, ovule, pollen and flower number. Furthermore, we wanted to test whether populations had spread from single origins or whether the dispersal is more erratic by comparing geographic and genetic distances following the approach by Genton *et al.*[30]. To examine these relationships, Spearman correlations were investigated using SPSS v. 15 [82].

## Competing interests

The authors declare that they have no competing interests.

## Authors’ contributions

RS carried out the molecular genetic studies, the crossing experiments, the investigations of floral morphology, seed germination and viability and performed most of the statistical analyses under the supervision of DA. RS and DA drafted the manuscript together. All authors read and approved the final manuscript.

## Supplementary Material

Additional file 1 **Information on the origin of samples used in the study.** The population in bold is the population which did not produce flowers during our crossing experiment. Populations in italics are the populations used for pollen and ovule counts. Individuals crossed refers to the number of individuals used in the crossing experiment. Notes refer to the number of investigated flowers (for mutants), buds, capsules and seeds. ^1^ = years obtained from literature [44,45]; ^2^ = years obtained from observations of local botanists (M. Thiv, Stuttgart, pers. comm.); 1^st^ obs.= date of the first observations in the introduced area; Pop. area = population size estimation; Cauc. - Caucasus, Sam.-Jav. - Samtskhe-Javakehti. Click here for file

Additional file 2 **Relationship between genetic and geographic distances within twenty introduced populations of *V. filiformis.*** Spearman correlation between genetic and geographic distances gave r = 0.2925, based on 190 comparisons in the German transect, p < 0.0001. Click here for file

Additional file 3 **Morphology of normal and abnormal flowers in *V. filiformis.*** A. Normal and radial flower with four petals, two equal stamens and one style. B. Normal flower conserved in alcohol. C. Androecium-mutant flowers observed in the introduced area (C_1_. Flower with one long and one short filament, C_2_. Flower without filament, C_3_. Flower with one stamen, C_4_. Flower without stamen, C_5_. Flower with three stamens). The scale corresponds to a length of 1 mm; d = dorsal; b = basal. Click here for file

Additional file 4 **Pollen and ovule production in*V. filiformis.*** N_flowers_ = number of flowers dissected; Pollen = pollen number; Ovule = ovule number; S.D. = standard deviation; % S.D. = percentage of the standard deviation. The uncapitalized color names correspond to the different crossing groups (Cros. group) whereas the capitalized color names correspond to the genetic clusters (Gen. group), as presented in Figures 2 and 3. Click here for file

Additional file 5 **Results from nested ANOVA for A) pollen data and B) ovule data.** d.f. = degree of freedom; SS = sum of squares; MSS = mean sum of squares; Significance level: * = P ≤ 0.05, ** = P ≤ 0.01. Click here for file

Additional file 6 **Seed size and seed number per capsule in *V. filiformis.*** N_caps._ = number of capsules collected in the native area or obtained during the crossing experiment; N_Seeds/Caps._ = number of seeds per capsule and per population; S.D. = standard deviation; % S.D. = percentage of the standard deviation; Total N_seed_ = total number of seeds measured per population. Seed length and width were measured under a graduated microscope and converted to mm. Click here for file

Additional file 7 **Relationships between the percentage of androecium mutant-flower, pollen and ovule number and genetic data.** A. Relationship between the percentage of androecium mutant-flowers per population and pollen production. B. Relationship between the percentage of androecium mutant-flowers per population and ovule production. C_1_. Relationship between pollen production and the number of polymorphic AFLP-fragment per population. C_2_. Relationship between pollen production and the number of fixed AFLP-fragment per population. D_1_. Relationship between the number of clones per population and pollen production. D_2_. Relationship between the number of clones per population and seed number per capsule according to maternal population. E_1_. Relationship between the number of polymorphic AFLP-fragment per population and the percentage of androecium mutant-flowers. E_2_. Relationship between the number of polymorphic AFLP-fragment per population and the flower number. Spearman correlations are indicated for each relationship as “R^2^ =”. Click here for file
