# Supplementary material for: Degradation of sexual reproduction in Veronica filiformis after introduction to Europe
Source: BMC Evol Biol. 2012 Dec 3;12:233. doi: 10.1186/1471-2148-12-233 (PMC3539859; doi:10.1186/1471-2148-12-233)
Supplement: Additional file 1 — Information on the origin of samples used in the study. The population in bold is the population which did not produce flowers during our crossing experiment. Populations in italics are the populations used for pollen and ovule counts. Individuals crossed refers to the number of individuals used in the crossing experiment. Notes refer to the number of investigated flowers (for mutants), buds, capsules and seeds. 1 = years obtained from literature [44,45]; 2 = years obtained from observations of local botanists (M. Thiv, Stuttgart, pers. comm.); 1st obs.= date of the first observations in the introduced area; Pop. area = population size estimation; Cauc. - Caucasus, Sam.-Jav. - Samtskhe-Javakehti. [file 1471-2148-12-233-S1.doc]

**Additional file 1 - Information on the origin of samples used in the study**

The population in bold is the population which did not produce flowers during our crossing experiment. Populations in italics are the populations used for pollen and ovule counts. Individuals crossed refers to the number of individuals used in the crossing experiment. Notes refer to the number of investigated flowers (for mutants), buds, capsules and seeds.

^1^ = years obtained from literature [44, 45]; ^2^ = years obtained from observations of local botanists (M. Thiv, Stuttgart, pers. comm.); 1^st^ obs. = date of the first observations in the introduced area; Pop. area = population size estimation; Cauc. - Caucasus, Sam.-Jav. - Samtskhe-Javakehti.

| **Population** | **Countries** | **Region** | | | **Code** | | **Coordinates (N, E)** | | | **1_st_ obs.** | **Pop. Area** | **Habitat** | **Indiv. crossed** | **Notes** |
| --- | --- | --- | --- | --- | --- | --- | --- | --- | --- | --- | --- | --- | --- | --- |
| Native populations |  | | |  | | | | | | | | | | |
| Uzungöl | Turkey | Pontic Mts., Prov. Trabzon | | | UzT | | N 40°37'18'' | E 040°16'50'' | | --- | large (<1600m²) | Plateau in the Anatolian mountains |  |  |
| *Mleta* | Georgia | Greater Cauc.-Kazbegi | | | Kz2 | | N 42°26'43'' | E 044°29'15'' | | --- | large (<1600m²) | Plateau in the Caucasian moutains (Greater) |  | 91 flowers, 10 buds |
| Goudaouri | Georgia | Greater Cauc.-Kazbegi | | | Kz4 | | N 42°28'30'' | E 044°28'39'' | | --- | large (<1600m²) | Plateau in the Caucasian moutains (Greater) |  | 254 flowers |
| *Cross Pass* | Georgia | Greater Cauc.-Kazbegi | | | Kz8 | | N 42°32'14'' | E 044°28'38'' | | --- | large (<1600m²) | Plateau in the Caucasian moutains (Greater) |  | 202 (3) flowers, 10 buds |
| *Kazbegi-Kobi* | Georgia | Greater Cauc.-Kazbegi | | | Kz9 | | N 42°32'41'' | E 044°29'28'' | | --- | large (<1600m²) | Plateau in the Caucasian mountains (Greater) |  | 189 flowers, 10 buds |
| Telavi | Georgia | Greater Cauc.-Kazbegi | | | Lg1 | | N 41°54'26'' | E 045°22'45'' | | --- |  |  |  | 38 seeds |
| Tetritslebi | Georgia | Greater Cauc.-Kazbegi | | | Lg3 | | N 41°51'45'' | E 045°19'02'' | | --- |  |  |  | 6 capsules, 36 seeds |
| Telavi-Gombori A | Georgia | Greater Cauc.-Kazbegi | | | Lg4 | | N 41°52'08'' | E 045°17'00'' | | --- |  |  |  | 5 capsulses, 43 seeds |
| Telavi-Gombori B | Georgia | Greater Cauc.-Kazbegi | | | Lg5 | | N 41°52'12'' | E 045°18'18'' | | --- |  |  |  | 1 capsule |
| Telavi-Gombori C | Georgia | Greater Cauc.-Kazbegi | | | Lg6 | | N 41°52'17'' | E 045°20'53'' | | --- |  |  |  | 3 capsules, 29 seeds |
| Telavi-Gombori D | Georgia | Greater Cauc.-Kazbegi | | | Lg7 | | N 41°52'40'' | E 045°16'43'' | | --- |  |  |  | 11 capsules, 78 seeds |
| Telavi-Gombori E | Georgia | Greater Cauc.-Kazbegi | | | Lg8 | | N 41°52'30'' | E 045°14'56'' | | --- |  |  |  | 2 capsules 13 seeds |
| Borjomi | Georgia | Lesser Cauc.-Sam.-Jav. | | | Bk2 | | N 41°48'45'' | E 043°26'25'' | | --- |  |  |  | 7 capsules, 34 seeds |
| Tsagueri, river Gujarula | Georgia | Lesser Cauc.-Sam.-Jav. | | | Bk3 | | N 41°48'25'' | E 043°28'09'' | | --- |  |  |  | 3 capsules |
| Tsagueri | Georgia | Lesser Cauc.-Sam.-Jav. | | | Bk4 | | N 41°47'48'' | E 043°28'20'' | | --- |  |  |  | 4 capsules, 17 seeds |
| Pataratsemi | Georgia | Lesser Cauc.-Sam.-Jav. | | | Bk5 | | N 41°47'40'' | E 043°28'05'' | | --- |  |  |  | 2 capsules, 17 seeds |
| Bakuriani A | Georgia | Lesser Cauc.-Sam.-Jav. | | | Bk7 | | N 41°44'09'' | E 043°31'03'' | | --- |  |  |  | 43 seeds |
| Bakuriani B | Georgia | Lesser Cauc.-Sam.-Jav. | | | Bk8 | | N 41°43'54'' | E 043°30'21'' | | --- |  |  |  | 15 seeds |
| Bakuriani-Tskratskaro | Georgia | Lesser Cauc.-Sam.-Jav. | | | Bk10 | | N 41°38'17'' | E 042°22'31'' | | --- | large (<1600m²) | Plateau in the Caucasian mountains (Lesser) |  |  |
| Agara | Georgia | Lesser Cauc.-Sam.-Jav. | | | Bk11 | | N 41°41'40'' | E 043°07'42'' | | --- | large (<1600m²) | Plateau in the Caucasian moutains (Lesser) |  | 1174 flowers |
| Batumi-Batskana | Georgia | Lesser Cauc.-Adjara | | | Bt1 | | N 41°39'01'' | E 041°40'02'' | | --- |  |  |  | 36 seeds |
| *Khulo* | Georgia | Lesser Cauc.-Adjara | | | Bt10 | | N 41°38'17'' | E 042°22'31'' | | --- | large (<1600m²) | Plateau in the Caucasian moutains (Lesser) |  | 171 flowers, 5 buds, 6 capsules, 59 seeds |
| *Chuasopeli* | Georgia | Lesser Cauc.-Adjara | | | Bt11 | | N 41°38'25'' | E 042°27'30'' | | --- | large (<1600m²) | Plateau in the Caucasian moutains (Lesser) |  | 55 flowers, 8 buds, 2 capsules, 14 seeds |
| Batumi | Georgia | Lesser Cauc.-Adjara | | | Bt12 | | N 41°38'01'' | E 042°29'49'' | | --- |  |  |  | 1 capsule |
| Introduced populations | | |  | | |  | | |  | | | | | |
| Tübingen | Germany | Baden-Württemberg | | | Tp | | N 48°31'36'' | E 009°02'11'' | | ? | small (<5m²) | Private garden, West Tübingen | 3 |  |
| *Tübingen* | Germany | Baden-Württemberg | | | Tb | | N 48°31'33'' | E 009°05'12'' | | 1909 ^1^ | small (<5m²) | Old botanical garden, Central Tübingen | 11 |  |
| Tübingen | Germany | Baden-Württemberg | | | Tl | | N 48°31'26'' | E 009°03'29'' | | 1909 ^1^ | large (<750m²) | Private collective garden, East Tübingen | 18 |  |
| Kirchentellinsfurt | Germany | Baden-Württemberg | | | Kt | | N 48°32'01'' | E 009°08'17'' | | ? | small (<5m²) | Grass close to a footpath | 13 |  |
| *Pliezhausen* | Germany | Baden-Württemberg | | | Pl | | N 48°33'03'' | E 009°11'02'' | | 1988 ^2^ | large (<1600m²) | Grass close to a footpath | 6 |  |
| Betzingen | Germany | Baden-Württemberg | | | Bz | | N 48°29'58'' | E 009°10'17'' | | ? | intermediate (<25m²) | Old cemetery of Reutlingen | 9 |  |
| *Reutlingen* | Germany | Baden-Württemberg | | | Re | | N 48°28'38'' | E 009°11'09'' | | 1987 ^2^ | large (<1600m²) | Parc of Schlattwiesen | 6 |  |
| Hohenwittlingen | Germany | Baden-Württemberg | | | Hw | | N 48°28'17'' | E 009°25'18'' | | 1984 ^2^ | small (<5m²) | Fruit tree culture, East Bad Urach | 4 |  |
| *Münsingen* | Germany | Baden-Württemberg | | | Mü | | N 48°24'39'' | E 009°29'28'' | | 2001 ^2^ | intermediate (<25m²) | Grass close to the train station | 15 |  |
| *Mehrstetten* | Germany | Baden-Württemberg | | | Mt | | N 48°22'31'' | E 009°33'47'' | | 2001 ^2^ | large (<300m²) | Private garden | 12 | 54 (3) flowers |
| Blaubeuren | Germany | Baden-Württemberg | | | Bl | | N 48°24'52'' | E 009°47'24'' | | 1975 ^2^ | small (<5m²) | Grass close to a school | 5 |  |
| Ulm | Germany | Bavaria | | | U | | N 48°23'38'' | E 009°59'16'' | | 2003 ^2^ | large (<500m²) | Old Botanical Garden of Ulm | 5 |  |
| *Wiblingen* | Germany | Bavaria | | | Wi | | N 48°21'18'' | E 009°59'04'' | | 1936 ^1^ | large (<500m²) | Old cemetery of Ulm | 7 |  |
| *Illerzell* | Germany | Bavaria | | | Il | | N 48°17'44'' | E 010°03'24'' | | 2003 ^2^ | large (<500m²) | Private collective garden | 2 | 103 (47) flowers |
| *Günzburg* | Germany | Bavaria | | | Gü | | N 48°27'14'' | E 010°16'38'' | | ? | small (<5m²) | Public garden in front of the castel | 5 |  |
| Burgau | Germany | Bavaria | | | Bg | | N 48°25'32'' | E 010°25'08'' | | ? | intermediate (<25m²) | Private garden | 5 |  |
| Ziemetshausen | Germany | Bavaria | | | Zi | | N 48°17'28'' | E 010°31'54'' | | ? | small (<5m²) | Private garden | 5 |  |
| **Großaitingen** | Germany | Bavaria | | | Gß | | N 48°13'56'' | E 010°46'51'' | | ? | small (<5m²) | Grass close to a footpath | - |  |
| Westheim | Germany | Bavaria | | | We | | N 48°23'02'' | E 010°48'59'' | | ? | intermediate (<25m²) | Grass close to a school | 3 |  |
| Augsburg | Germany | Bavaria | | | A | | N 48°22'38'' | E 010°54'01'' | | 1939 ^1^ | intermediate (<25m²) | Public garden, Stephingergraben | 3 |  |
| Bonn-Rheinaue | Germany | Northrhine-Westfalia | | | Bo | | N 50°42'44'' | E 007°08'17'' | | ? | large (<1600m²) | Public garden along the Rhein river |  |  |
| Mainz | Germany | Rhineland-Palatia | | | Mz | | N 49°59'24'' | E 008°14'22'' | | ? | intermediate (<25m²) | Arboretum, Botanical Garden |  |  |
