# Supplementary figures and images for: Degradation of sexual reproduction in Veronica filiformis after introduction to Europe
Source: BMC Evol Biol. 2012 Dec 3;12:233. doi: 10.1186/1471-2148-12-233 (PMC3539859; doi:10.1186/1471-2148-12-233)

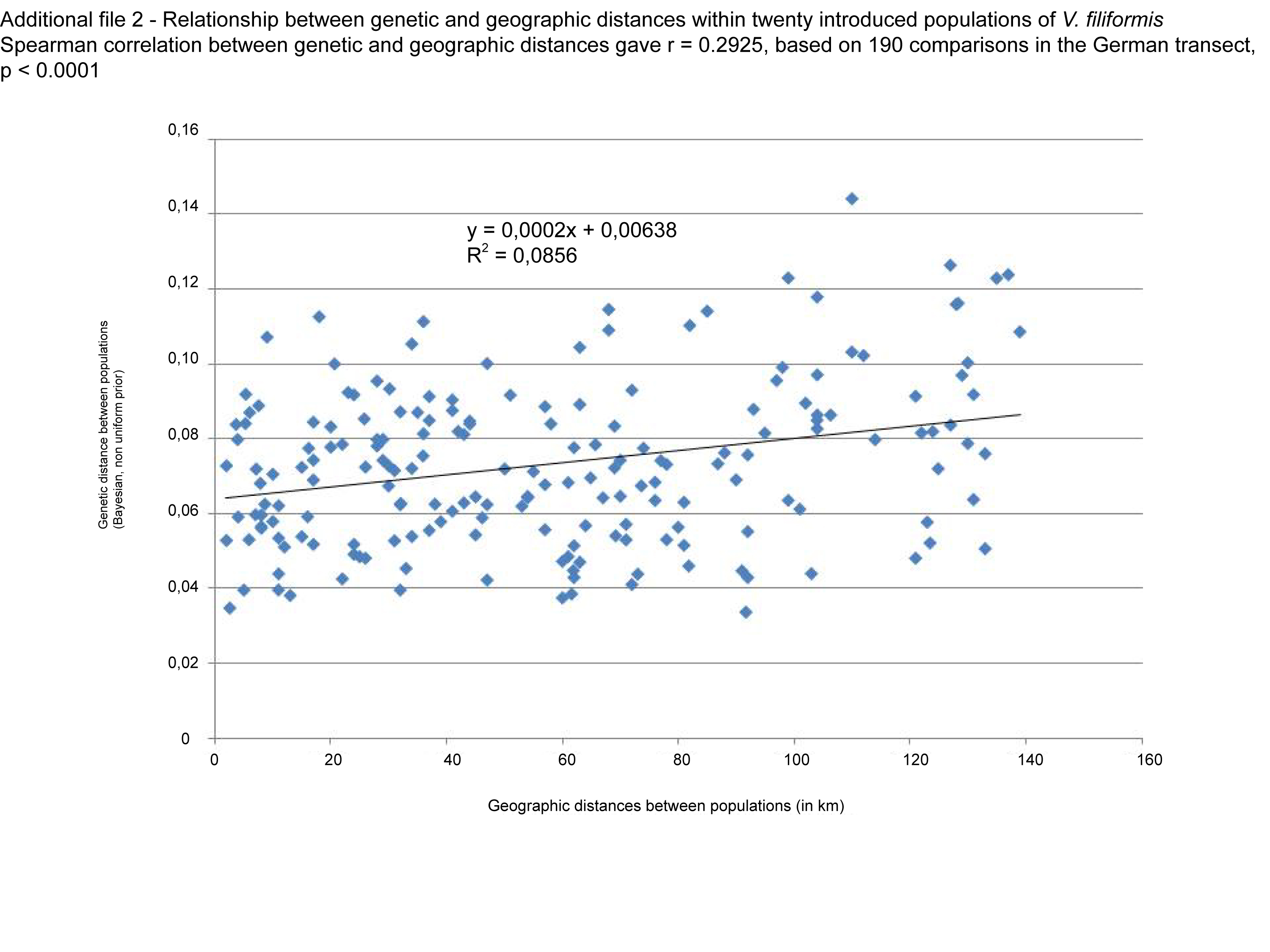

Supplement: Additional file 2 — Relationship between genetic and geographic distances within twenty introduced populations of V. filiformis. Spearman correlation between genetic and geographic distances gave r = 0.2925, based on 190 comparisons in the German transect, p < 0.0001. [file 1471-2148-12-233-S2.jpeg]

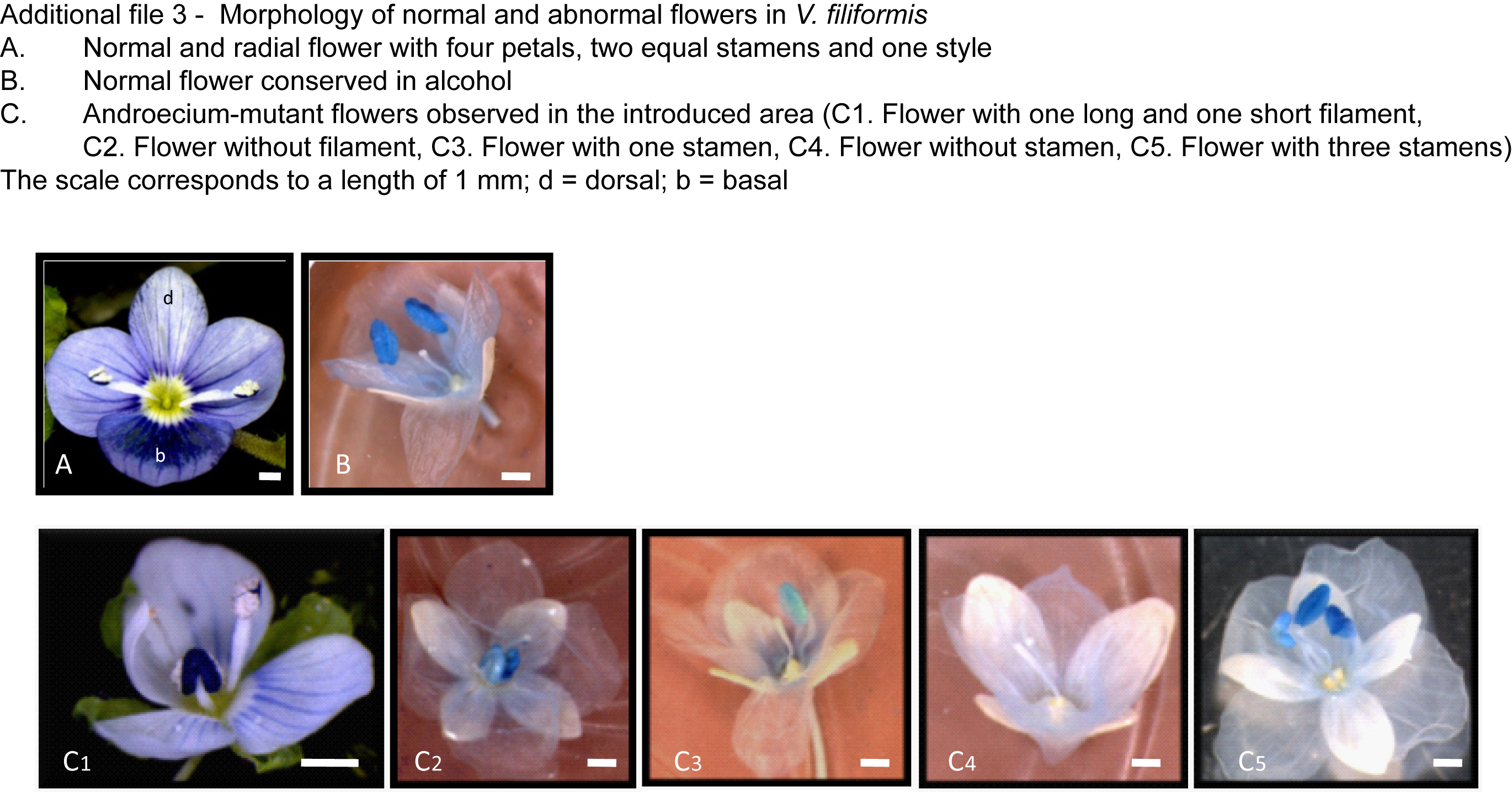

Supplement: Additional file 3 — Morphology of normal and abnormal flowers in V. filiformis. A. Normal and radial flower with four petals, two equal stamens and one style. B. Normal flower conserved in alcohol. C. Androecium-mutant flowers observed in the introduced area (C1. Flower with one long and one short filament, C2. Flower without filament, C3. Flower with one stamen, C4. Flower without stamen, C5. Flower with three stamens). The scale corresponds to a length of 1 mm; d = dorsal; b = basal. [file 1471-2148-12-233-S3.jpeg]

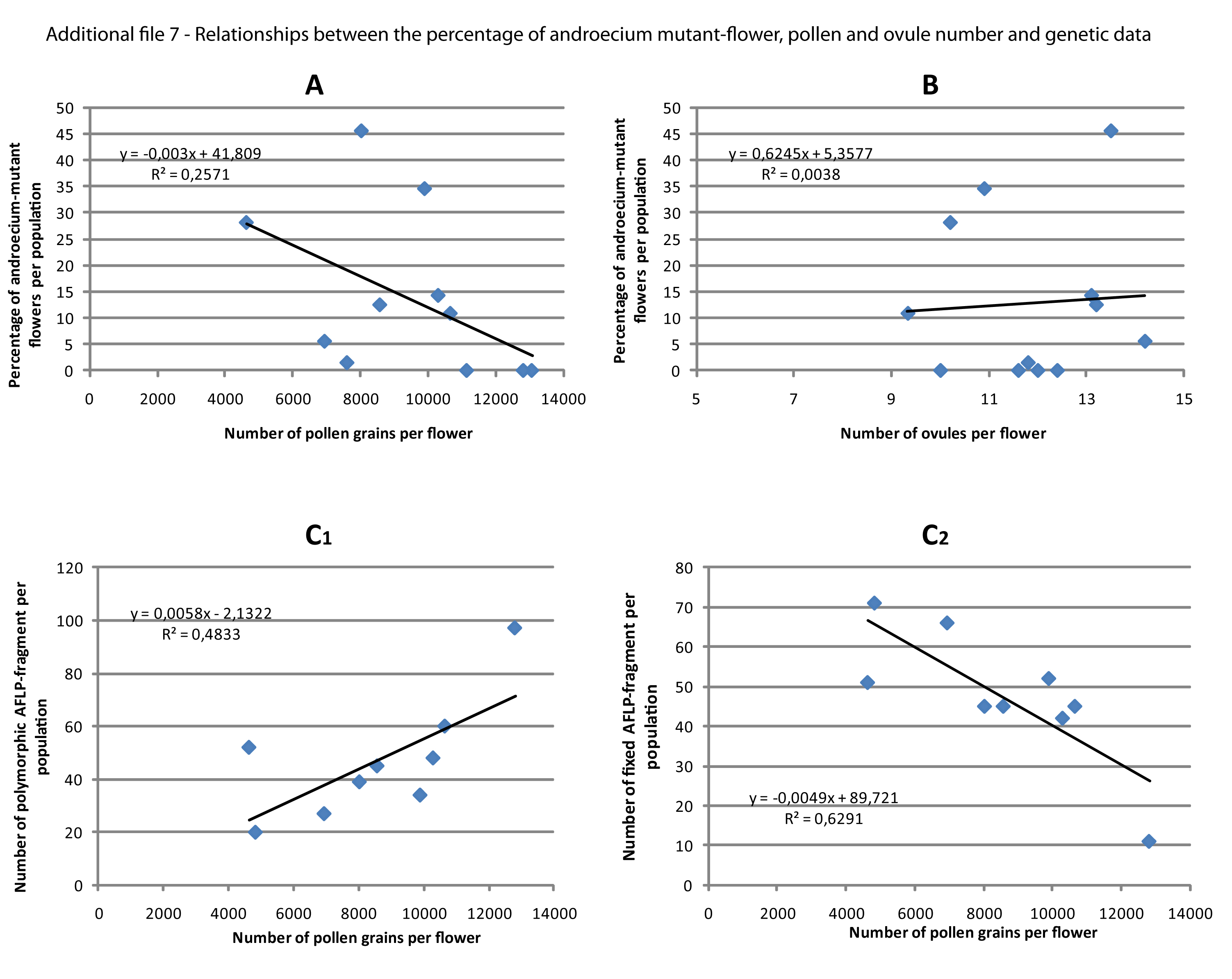

Supplement: Additional file 7 — Relationships between the percentage of androecium mutant-flower, pollen and ovule number and genetic data. A. Relationship between the percentage of androecium mutant-flowers per population and pollen production. B. Relationship between the percentage of androecium mutant-flowers per population and ovule production. C1. Relationship between pollen production and the number of polymorphic AFLP-fragment per population. C2. Relationship between pollen production and the number of fixed AFLP-fragment per population. D1. Relationship between the number of clones per population and pollen production. D2. Relationship between the number of clones per population and seed number per capsule according to maternal population. E1. Relationship between the number of polymorphic AFLP-fragment per population and the percentage of androecium mutant-flowers. E2. Relationship between the number of polymorphic AFLP-fragment per population and the flower number. Spearman correlations are indicated for each relationship as “R2 =”. [file 1471-2148-12-233-S7.jpeg]
