# Supplementary material for: Degradation of sexual reproduction in Veronica filiformis after introduction to Europe
Source: BMC Evol Biol. 2012 Dec 3;12:233. doi: 10.1186/1471-2148-12-233 (PMC3539859; doi:10.1186/1471-2148-12-233)
Supplement: Additional file 4 — Pollen and ovule production inV. filiformis. Nflowers = number of flowers dissected; Pollen = pollen number; Ovule = ovule number; S.D. = standard deviation; % S.D. = percentage of the standard deviation. The uncapitalized color names correspond to the different crossing groups (Cros. group) whereas the capitalized color names correspond to the genetic clusters (Gen. group), as presented in Figures 2 and 3. [file 1471-2148-12-233-S4.docx]

| **Area** | **Region** | **Population** | **Code** | **N_flowers_** | **Pollen** | **S.D.** | **% S.D.** | **Ovule** | **S.D.** | **% S.D.** | **Cros. group** | **Gen. group** |
| --- | --- | --- | --- | --- | --- | --- | --- | --- | --- | --- | --- | --- |
|  |  |  |  |  |  |  |  |  |  |  |  |  |
| **Native** | | | Nat. | 43 | 11500 | 2309 | 20,10% | 11,6 | 0,9 | 8,00% | --- | --- |
|  |  |  |  |  |  |  |  |  |  |  |  |  |
|  | Kazbegi | | GC | 30 | 11160 | 4512 | 40,43% | 11,9 | 1,4 | 12,04% | --- | --- |
|  |  | Mleta | Kz2 | 10 | 13060 | 4471 | 34,20% | 12,4 | 1,3 | 10,20% | --- | --- |
|  |  | Cross Pass | Kz8 | 10 | 7610 | 4097 | 53,80% | 11,8 | 1,8 | 14,80% | --- | --- |
|  |  | Kazbegi-Kobi | Kz9 | 10 | 12810 | 2788 | 21,80% | 11,6 | 1,3 | 10,90% | --- | --- |
|  |  |  |  |  |  |  |  |  |  |  |  |  |
|  | Adjara |  | LC | 13 | 12259 | 5248 | 42,81% | 11,1 | 1,8 | 15,87% | --- | --- |
|  |  | Khulo | Bt10 | 5 | 11140 | 3035 | 27,20% | 12,0 | 0,0 | 0,00% | --- | --- |
|  |  | Chuasopeli | Bt11 | 8 | 12881 | 4223 | 34,20% | 10,0 | 2,3 | 23,10% | --- | --- |
|  |  |  |  |  |  |  |  |  |  |  |  |  |
|  |  |  |  |  |  |  |  |  |  |  |  |  |
| **Introduced** | | | Intr. | 92 | 7988 | 2350 | 29,40% | 12,0 | 1,8 | 14,60% | 4 | 4 |
|  |  |  |  |  |  |  |  |  |  |  |  |  |
|  | Baden-Württemberg | | BW | 48 | 7102 | 3610 | 50,82% | 12,0 | 2,3 | 18,88% | --- | --- |
|  |  | Tübingen | Tb | 5 | 10658 | 2507 | 23,50% | 9,3 | 1,7 | 17,90% | blue | Blue |
|  |  | Pliezhausen | Pl | 15 | 4641 | 2405 | 51,80% | 10,2 | 1,5 | 15,00% | blue | Blue |
|  |  | Reutlingen | Re | 10 | 4840 | 2459 | 50,80% | 13,0 | 1,3 | 10,30% | green | Pink |
|  |  | Münsingen | Mü | 8 | 9900 | 3874 | 39,10% | 10,9 | 1,2 | 11,50% | pink | Green / Red |
|  |  | Mehrstetten | Mt | 10 | 6950 | 3194 | 46,00% | 14,2 | 2,7 | 19,30% | violet | Red |
|  |  |  |  |  |  |  |  |  |  |  |  |  |
|  | Bavaria |  | BV | 44 | 9080 | 2514 | 27,68% | 13,1 | 1,4 | 10,65% | --- | --- |
|  |  | Wiblingen | Wi | 10 | 8578 | 2604 | 30,40% | 13,4 | 1,9 | 14,30% | green | Pink |
|  |  | Illerzell | Il | 9 | 8035 | 2720 | 33,90% | 13,5 | 2,1 | 15,70% | green | Red |
|  |  | Günzburg | Gü | 25 | 10299 | 2393 | 23,20% | 12,9 | 1,1 | 8,10% | green | Red |

**Additional file 4 - Pollen and ovule production in *V. filiformis***

N_flowers_ = number of flowers dissected; Pollen = pollen number; Ovule = ovule number; S.D. = standard deviation; % S.D. = percentage of the standard deviation. The names of colors starting without capital letter correspond to the different crossing groups (Cros. group) whereas the names starting with capital letter correspond to the genetic clusters (Gen. group), as presented in Figures 2 and 3.
