# Supplementary material for: Degradation of sexual reproduction in Veronica filiformis after introduction to Europe
Source: BMC Evol Biol. 2012 Dec 3;12:233. doi: 10.1186/1471-2148-12-233 (PMC3539859; doi:10.1186/1471-2148-12-233)
Supplement: Additional file 5 — Results from nested ANOVA for A) pollen data and B) ovule data. d.f. = degree of freedom; SS = sum of squares; MSS = mean sum of squares; Significance level: * = P ≤ 0.05, ** = P ≤ 0.01. [file 1471-2148-12-233-S5.doc]

**Additional file 5 - Results from nested ANOVA for A) pollen data and B) ovule data.**

d.f. = degree of freedom; SS = sum of squares; Significance level: * = P ≤ 0.05, ** = P ≤ 0.01

A

|  | d.f. | SS | F | Probability |
| --- | --- | --- | --- | --- |
| Area | 1 | 356679680 | 32.154 | <0.001 ** |
| Crossing group | 3 | 96324645 | 2.895 | 0.038 * |
| Population | 8 | 473067124 | 5.331 | <0.001 ** |

B

|  | d.f. | SS | F | Probability |
| --- | --- | --- | --- | --- |
| Area | 1 | 8.41 | 2.334 | 0.130 |
| Crossing group | 3 | 155.15 | 14.363 | <0.001 ** |
| Population | 8 | 22.29 | 0.774 | 0.627 |
