# Supplementary material for: Degradation of sexual reproduction in Veronica filiformis after introduction to Europe
Source: BMC Evol Biol. 2012 Dec 3;12:233. doi: 10.1186/1471-2148-12-233 (PMC3539859; doi:10.1186/1471-2148-12-233)
Supplement: Additional file 6 — Seed size and seed number per capsule in V. filiformis. Ncaps. = number of capsules collected in the native area or obtained during the crossing experiment; NSeeds/Caps. = number of seeds per capsule and per population; S.D. = standard deviation; % S.D. = percentage of the standard deviation; Total Nseed = total number of seeds measured per population. Seed length and width were measured under a graduated microscope and converted to mm. [file 1471-2148-12-233-S6.docx]

| **Area / Region** | **Population** | **Code** | **N_caps._** | **N_Seeds/Caps._** | **S.D.** | **% S.D.** | **Total N_Seed_** | **Length** | **Width** | **Cros. group** | **Gen. group** |
| --- | --- | --- | --- | --- | --- | --- | --- | --- | --- | --- | --- |
|  |  |  |  |  |  |  |  |  |  |  |  |
| **Native** |  | Nat. | 53 | 6.3 | 2.4 | 38.17% | 472 | 13.4 | 9.3 | --- | --- |
|  |  |  |  |  |  |  |  |  |  |  |  |
| **Greater Caucasus** | | GC | 28 | 6.6 | 2.3 | 34.85% | 237 | 13.3 | 9.3 | --- | --- |
| **Kakheti** | Telavi | Lg1 | --- | --- | --- | --- | 38 | 11.1 | 8.2 | --- | --- |
|  | Tetritslebi | Lg3 | 6 | 6.0 | 2.4 | 40.00% | 36 | 13.8 | 9.6 | --- | --- |
|  | Telavi-Gombori A | Lg4 | 5 | 8.0 | 1.2 | 15.00% | 43 | 14.6 | 10.5 | --- | --- |
|  | Telavi-Gombori B | Lg5 | 1 | 5.0 | 0.0 | 0.00% | --- | --- | --- | --- | --- |
|  | Telavi-Gombori C | Lg6 | 3 | 7.0 | 1.7 | 24.29% | 29 | 13.9 | 10.1 | --- | --- |
|  | Telavi-Gombori D | Lg7 | 11 | 6.5 | 2.7 | 41.54% | 78 | 13.3 | 8.7 | --- | --- |
|  | Telavi-Gombori E | Lg8 | 2 | 6.5 | 3.5 | 53.85% | 13 | 13.0 | 9.9 | --- | --- |
|  |  |  |  |  |  |  |  |  |  |  |  |
| **Lesser Caucasus** | | LC | 25 | 6.0 | 2.6 | 42.67% | 235 | 13.4 | 9.3 | --- | --- |
| **Adjara** | Batumi-Batskana | Bt1 | --- | --- | --- | --- | 36 | 13.9 | 10.0 | --- | --- |
|  | Khulo | Bt10 | 6 | 7.7 | 1.2 | 15.71% | 59 | 13.9 | 9.5 | --- | --- |
|  | Chuasopeli | Bt11 | 2 | 7.0 | 0.0 | 0.00% | 14 | 14.4 | 9.2 | --- | --- |
|  | Batumi | Bt12 | 1 | 10.0 | 0.0 | 0.00% | --- | --- | --- | --- | --- |
| **Samtskhe-** | Borjomi | Bk2 | 7 | 4.9 | 2.4 | 49.18% | 34 | 14.7 | 10.8 | --- | --- |
| **Javakhéti** | Tsagueri, r. Gujarula | Bk3 | 3 | 4.0 | 2.6 | 66.00% | --- | --- | --- | --- | --- |
|  | Tsagueri | Bk4 | 4 | 4.3 | 2.6 | 61.16% | 17 | 13.5 | 9.5 | --- | --- |
|  | Pataratsemi | Bk5 | 2 | 8.5 | 0.7 | 8.35% | 17 | 12.0 | 8.3 | --- | --- |
|  | Bakuriani A | Bk7 | --- | --- | --- | --- | 43 | 11.0 | 7.4 | --- | --- |
|  | Bakuriani B | Bk8 | --- | --- | --- | --- | 15 | 14.7 | 9.7 | --- | --- |
|  |  |  |  |  |  |  |  |  |  |  |  |
| **Introduced** | | Intr. | 83 | 7.4 | 2.8 | 37.33% | 690 | 11.2 | 8.3 | --- | --- |
|  |  |  |  |  |  |  |  |  |  |  |  |
| **Baden-Württemberg** | | BW | 75 | 7.5 | 2.7 | 36.17% | 620 | 11.2 | 8.2 | --- | --- |
|  | Tübingen | Tb | 5 | 7.2 | 1.3 | 18.06% | 42 | 9.3 | 6.5 | blue | Blue |
|  | Tübingen | Tl | 26 | 9.3 | 2.1 | 22.14% | 246 | 11.2 | 8.0 | red | Green |
|  | Kirchentellinsfurt | Kt | 8 | 8.3 | 2.6 | 31.52% | 66 | 11.5 | 8.3 | green | Turquoise |
|  | Pliezhausen | Pl | 18 | 5.5 | 2.3 | 42.36% | 99 | 12.0 | 8.9 | blue | Blue |
|  | Reutlingen | Re | 4 | 7.3 | 1.9 | 26.07% | 29 | 9.7 | 7.1 | green | Pink |
|  | Münsingen | Mü | 4 | 7.3 | 3.3 | 45.52% | 52 | 11.4 | 8.2 | pink | Green / Red |
|  | Mehrstetten | Mt | 10 | 6.4 | 3.0 | 47.27% | 86 | 11.7 | 9.1 | violet | Red |
|  |  |  |  |  |  |  |  |  |  |  |  |
| **Bavaria** |  | BV | 8 | 6.5 | 3.3 | 50.00% | 70 | 11.1 | 9.0 | --- | --- |
|  | Ulm | U | 1 | 2.0 | 0.0 | 0.00% | --- | --- | --- | green | Pink |
|  | Wiblingen | Wi | 2 | 3.5 | 0.7 | 20.20% | 10 | 9.9 | 7.9 | green | Pink |
|  | Burgau | Bg | 2 | 9.0 | 0.0 | 0.00% | 18 | 10.0 | 8.3 | green | Pink |
|  | Ziemetshausen | Zi | 2 | 9.5 | 2.1 | 22.32% | 30 | 11.7 | 9.5 | green | Red |
|  | Augsburg | A | 1 | 6.0 | 0.0 | 0.00% | 12 | 12.4 | 9.5 | green | Turquoise |

**Additional file 6 - Seed size and seed number per capsule in *V. filiformis***

N_caps._ = number of capsules collected in the native area or obtained during the crossing experiment; N_Seeds/Caps._ = number of seeds per capsule and per population; S.D. = standard deviation; % S.D. = percentage of the standard deviation; Total N_seed_ = total number of seeds measured per population. Seed length and width were measured under a graduated microscope and converted to mm.
